# Molecular mechanism of a coastal cyanobacterium *Synechococcus* sp. PCC 7002 adapting to changing phosphate concentrations

**DOI:** 10.1007/s42995-024-00244-y

**Published:** 2024-07-22

**Authors:** Qiao-Wei Sun, Yu Gao, Jordan Wang, Fei-xue Fu, Cheng-Wen Yong, Shuang-Qing Li, Hai-Long Huang, Wei-Zhong Chen, Xin-Wei Wang, Hai-Bo Jiang

**Affiliations:** 1 School of Marine Sciences, Ningbo University, Ningbo, 315211 China; 2 Southern Marine Science and Engineering Guangdong Laboratory (Zhuhai), Zhuhai, 519080 China; 3 School of Life Sciences, Central China Normal University, Wuhan, 430079 China; 4 Department of Biological Sciences, University of Southern California, Los Angeles, CA 90089 USA

**Keywords:** *Synechococcus* sp. PCC 7002, Phosphorus fluctuation, Cyanobacteria, Gene knockout, Molecular mechanism

## Abstract

Phosphorus concentration on the surface of seawater varies greatly with different environments, especially in coastal. The molecular mechanism by which cyanobacteria adapt to fluctuating phosphorus bioavailability is still unclear. In this study, transcriptomes and gene knockouts were used to investigate the adaptive molecular mechanism of a model coastal cyanobacterium *Synechococcus* sp. PCC 7002 during periods of phosphorus starvation and phosphorus recovery (adding sufficient phosphorus after phosphorus starvation). The findings indicated that phosphorus deficiency affected the photosynthesis, ribosome synthesis, and bacterial motility pathways, which recommenced after phosphorus was resupplied. Even more, most of the metabolic pathways of cyanobacteria were enhanced after phosphorus recovery compared to the control which was kept in continuous phosphorus replete conditions. Based on transcriptome, 54 genes potentially related to phosphorus-deficiency adaptation were selected and knocked out individually or in combination. It was found that five mutants showed weak growth phenotype under phosphorus deficiency, indicating the importance of the genes (*A0076*, *A0549-50*, *A1094*, *A1320*, *A1895*) in the adaptation of phosphorus deficiency. Three mutants were found to grow better than the wild type under phosphorus deficiency, suggesting that the products of these genes (*A0079*, *A0340*, *A2284–86*) might influence the adaptation to phosphorus deficiency. Bioinformatics analysis revealed that cyanobacteria exposed to highly fluctuating phosphorus concentrations have more sophisticated phosphorus acquisition strategies. These results elucidated that *Synechococcus* sp. PCC 7002 have variable phosphorus response mechanisms to adapt to fluctuating phosphorus concentration, providing a novel perspective of how cyanobacteria may respond to the complex and dynamic environments.

**Supplementary Information:**

The online version contains supplementary material available at 10.1007/s42995-024-00244-y.

## Introduction

Phosphorus (P) is a key nutrient involved in substance synthesis and energy transfer in phytoplankton (Fei et al. 2018). The distribution and concentration of P in phytoplankton habitats are heterogeneous in both the vertical and horizontal directions. In general, the concentration of dissolved inorganic phosphorus (DIP) is low in the surface seawater, while it is relatively high in deep ocean (Lin et al. 2016). Coastal regions are located near terrestrial landmasses, resulting in a higher influx of exogenous phosphorus (Lin et al. 2016; Yuan et al. 2018). In contrast, several open-ocean regions experience P limitation, such as the especially in the North Atlantic Ocean (Lin et al. 2016). The distribution of dissolved organophosphorus in surface seawater is also heterogeneous, and its concentration is reported to be affected by inorganic phosphorus stress as well as iron stress (Liang et al. 2022). Previous studies have concluded that P concentration in upwelling regions can be altered up to 10 times the normal amount in the presence of monsoons and ocean currents (Mackey et al. 2012). Consequently, the uneven distribution and concentration of P in the ocean influences the interspecific competition and dispersion of phytoplankton (Viličić et al. 2011). For example, fluctuation in DIP concentration controls the changes in phytoplankton populations, and algal blooms frequently occur in nearshore waters due to eutrophication in the coastal region (Dyhrman and Ruttenberg 2006; Pitcher and Louw 2021).

Contributing to almost 25% of the global primary productivity and 50% of the net primary productivity in some sea areas, cyanobacteria are the most abundant unicellular class group among phytoplankton (Flombaum et al. 2013). To adapt to the changes in P concentration in their habitats, cyanobacteria have developed many adaptive strategies during their long-term evolution. In phosphate-deficient regions, cyanobacteria can alleviate P stress by reducing cell size, replacing membrane phospholipids, slowing cell growth, and forming symbiotic metabolic coupling with bacteria (Li et al. 2016; Mohlin and Wulff 2009; Yang et al. 2022; Zhang et al. 2015, 2018). Additionally, cyanobacteria may alleviate the stress caused by low P through utilizing different forms of P. For example, cyanobacteria can increase cellular alkaline phosphatase (AP) activity in P-limited environments or increase expression of the C–P lyase gene, *phnJ*, to utilize organic P (Lin et al. 2011; Sosa et al. 2019). After an increase in P concentration, cyanobacteria can increase P storage by excessive P uptake, which is known as the luxurious P uptake mechanism (Jentzsch et al. 2023).

Inorganic phosphate transport system (Pst system, including PstSCAB) and the two-component regulatory system are considered to play a vital role in adapting to the fluctuation of P concentration in cyanobacteria (Muñoz-Martín et al. 2011; Pereira et al. 2019). Histidine kinase-response regulator SphS–SphR has been identified in *Synechocystis* sp. PCC 6803 and *Synechococcus* sp. PCC 7942 (Mann and Scanlan 1994; Suzuki et al. 2004). In recent years, Pho box was found in the 5′ end of the C–P lyase of the thermophilic cyanobacteria *Synechococcus* OS-B′, which regulates the expression of downstream genes in a P concentration-dependent manner (Jin et al. 2021). Most of these studies were conducted in freshwater cyanobacterial species, which promotes understanding of how freshwater cyanobacteria acquire P and their response to P signals. However, there is a difference between marine and freshwater environments: marine cyanobacteria usually have more streamlined genomes compared to freshwater species. Especially in coastal waters, P environments have changed dramatically in recent years under the influence of human activities. Due to different P utilization strategies, various types of cyanobacteria respond differently to the changing coastal P environments, which have a profound impact on the community composition and distribution of the marine phytoplankton. So far, the molecular mechanisms used by cyanobacteria to adapt to varying P concentrations have not been clearly revealed.

*Synechococcus* sp. PCC 7002 is model coastal cyanobacterium that can be genetically manipulated. Previous studies have reported the transcriptome changes of *Synechococcus* sp. PCC 7002 under short-term P deficiency treatment (Ludwig and Bryant 2012), but little is known about how it adapts to fluctuating P concentration. To further explore the potential molecular mechanism of marine cyanobacteria coping with different environmental P concentrations, we compared the transcriptional levels of cyanobacteria under the conditions of standard P(CK), P deficiency (PL), and P recovery (PL_RS). A large-scale gene knockout approach was used to reveal the genes that allow *Synechococcus* sp. PCC 7002 to adapt to the changes of P concentration. Possible pathways of P acquisition in *Synechococcus* sp. PCC 7002 were proposed in this study. Furthermore, the distribution of P acquisition genes possessed by cyanobacteria in different habitats was analyzed by bioinformatics. The findings provide many useful information on the molecular mechanisms of marine phytoplankton respond to changing P concentrations.

## Materials and methods

### Cyanobacteria strains and culture conditions

The coastal cyanobacterium *Synechococcus* sp. PCC 7002, originated from Jindong Zhao’s lab (Peking University, China), was used in this study. All cyanobacteria were cultured in A^+^ medium with either liquid shaking culture or solid plate standing culture (reference for A^+^ medium). All solutions and media were sterilized by autoclave or vacuum filtration. A^+^ solid medium was supplemented with 0.4% Na_2_S_2_O_3_ and 1.2% agar. The culture conditions were as follows: 30℃, 110 r/min continuous light (40 µE·m^−2^·s^−1^) culture. The static culture conditions of solid plates were as follows: constant temperature at 30 °C and 30 µE·m^−2^·s^−1^ light intensity. The mutant strain grew under conditions corresponding to the resistance. The antibiotic spectinomycin (50 g/mL) was added to the liquid medium, and the concentration in the solid medium was 100 g/mL. In P deficiency experiments, we used normal A^+^ medium as the control (370 µmol/L KH_2_PO_4_) and 4 µmol/L KH_2_PO_4_ as the phosphate limiting condition for algal strains. To prevent K^+^ concentration from affecting experimental results, KCl was added to the P-deficient medium to equalize K^+^ concentrations with control cultures. Before P-deficient cultivation, cyanobacteria were grown in a standard medium until the logarithmic growth phase. Then, cells were washed twice in P-free A^+^ medium to remove extracellular phosphate.

### P starvation and P recovery experiments

The WT was cultured until the logarithmic growth phase, and then, cells were collected by centrifugation at 6000 r/min. Collected cells were washed twice with a P-free A^+^ liquid medium after which the cells were resuspended in P-free medium. The resuspended cells were inoculated in triplicates into A^+^ liquid medium with a standard P (370 µmol/L) concentration and P-free (0 µmol/L), and an initial OD_730_ of 0.02 was guaranteed for each replicate. For the CK group, samples were collected at standard P concentrations until the fourth day of culture. For the PL group, samples were collected by culturing under P-free conditions until the fourth day. As for the PL_RS group, cells were first cultured under P-free conditions, supplemented with KH_2_PO_4_ to the standard P concentration on the 10th day, and samples were collected on the 14th day. The cells were collected by centrifugation at 6000 r/min for a total of nine samples. The samples were flash frozen in liquid nitrogen and stored at − 80 °C refrigerator.

### RNA extraction and RNA-seq analysis

The samples snap-frozen in liquid nitrogen were removed, and the algal cells were subsequently poured into a mortar and ground with liquid nitrogen. After the cells were fully lysed using 3.5 mL Trizol (Invitrogen), 1.75 mL of lysate was aspirated into a new centrifuge tube and repeatedly blown. It was left on ice for 5 min and then centrifuged at 12,000 r/min for 5 min at 4 °C. After the supernatant was sucked, 400 µL chloroform solution was added, vortexed for 15 s, left on ice for 5 min, and centrifuged at 12,000 r/min for 15 min at 4 °C. The supernatant was aspirated, and an equal volume of isopropanol was added, mixed, and allowed to stand for 20 min at − 20 °C. The supernatant was discarded by centrifugation at 12,000 r/min for 15 min at 4 °C, and 1 mL of 75% ethanol was added to the precipitate for washing. After centrifugation for 3 min, the supernatant was discarded, and the operation was repeated once. They were then dried at 25 °C for 5 min to remove ethanol residue. After, 20 µL DEPC water was added to dissolve the RNA samples. Finally, total RNA content and quality were determined by agarose gel electrophoresis and NanoDrop (Thermo).

Library construction was performed by GENE READ (Wuhan, China) and sequencing was performed on the Illumina NovaSeq 6000. After, the raw data obtained was filtered to remove interference information and get Clean Data. Trimmomatic software (Bolger et al. 2014) was used for data filtering and FastQC software was used to evaluate the quality of the filtered data. The quality control data of each sample were compared with the reference genome sequence (*Synechococcus* sp. PCC 7002) using HISAT2 (2.2.1) software. The alignment results were analyzed using featureCounts in the subread (v2.0.1) software to count the number of reads covered from start to end for each gene (Liao et al. 2014). Fragments per kilobase of transcript per million fragments mapped (FPKM) was used to calculate the expression level of each gene using RSEM software. For differential expression, the count matrix of different comparison groups was calculated using the DESeq2 R package (1.20). The resulting *p* values were adjusted using the Benjamini and Hochberg’s false discovery rate (FDR) (Love et al. 2014). Genes with an adjusted p value < 0.05 and |log2 Fold Change (FC)|≥ 1 were considered differentially expressed genes (DEGs). Finally, cluster Profiler (3.8.1) software was mainly used for differential Gene enrichment analysis, including KEGG pathway enrichment analysis. The raw data reported in this paper have been deposited in the Genome Sequence Archive (Genomics, Proteomics & Bioinformatics 2023) in National Genomics Data Center (Nucleic Acids Res 2023), China National Center for Bioinformation/Beijing Institute of Genomics, Chinese Academy of Sciences (GSA: CRA014148) that are publicly accessible at https://ngdc.cncb.ac.cn/gsa.

### Construction of mutant strains

The mutant strains were obtained by the method of deletion mutation. Construction of recombinant plasmids was performed by restriction enzyme digestion and ligation (Jiang et al. 2015). Based on the pUC19-T vector (Takara), a spectomycin-resistant Ω fragment called pUC19-Ω was inserted. Then, two polyclonal fragments rich in endonuclease sites were inserted into the pUC19-T vector called pUC19-MCS-Ω. Next, *A0076*::Ω was used as an example to introduce the recombinant plasmid construction process. The A0076-1 fragment with restriction site Mlu I/EcoR V was amplified using the primers *A0076*-up-1/*A0076*-up-2 from the genomic DNA of WT *Synechococcus* sp. PCC 7002. The *A0076*-down fragment with the restriction site Bgl II/Xho I was amplified using primers *A0076*-dn-1/*A0076*-dn-2. The *A0076*-up fragment and vector pUC19-MCS-Ω were digested with restriction enzymes Mlu I and EcoR V, respectively, and the recombinant plasmid with the *A0076*-up fragment was obtained after fragment ligation. The *A0076*-down fragment was ligated to the recombinant plasmid in the same way used to obtain recombinant plasmids with *A0076*-up and *A0076*-down fragments. The obtained recombinant plasmid was transformed with the WT *Synechococcus* sp. PCC 7002, and the homozygous mutant Mut-A0076 was purified by continuous passage on resistant plates. Transformation of *Synechococcus* sp. PCC 7002 was performed as described by Williams (Williams 1988). The species used in this study are shown in Table S3. The primers used in this study are described in detail in Table S4.

### Determination of physiological parameters in mutant strains

The growth status of the algal strains was determined by measuring OD_730_, and the optical density (OD) was measured every 2 days until the 10th day. Three biological replicates were set for each algal strain. The initial inoculation OD_730_ was 0.02 for each strain. Growth rates were calculated according to the calculation formula: ln [OD_730_(day8)/OD_730_(day2)]/6. The content of chlorophyll a was determined by spectrophotometry. The value of OD_665_ was determined, and the content was calculated by the formula (Williams et al. 1988), Chl *a* (µg mL^−1^) = 12.6 × OD_665_. The FluorPen/AquaPen (EcoTech, China) was used to measure the *F*_*V*_*/F*_*M*_. Before the fluorescence measurement, the culture was subjected to 15 min of dark adaptation. The maximum quantum yield (*F*_*V*_*/F*_*M*_) was calculated on the basis of the equation (Fu et al. 2007): *F*_*V*_/*F*_*M*_ = (*F*_*M*_ − *F*_*0*_)/*F*_*M*_.

### Bioinformatics analysis

This article used the nucleic acid sequence and amino acid sequence from the KEGG database (https://www.kegg.jp/) and CyanoBase database (http://genome.microbedb.jp/cyanobase/). Using the CyanoOmicsDB website for homologous comparison (http://www.cyanoomics.cn/lz/index) (Xu et al. 2016). It is considered that there is no homologous protein with expected value (E value) > 1E−8. For results with E value > 1E−8, further confirmation will be performed on KEGG database and CyanoBase database. Heat map visualization was performed using TBTools software v.1.098765 (Chen et al. 2020; Zhou et al. 2022). The tools used for subcellular localization prediction include Gneg-mPLoc (http://www.csbio.sjtu.edu.cn/bioinf/Gneg-multi/), CELLO (http://cello.life.nctu.edu.tw/), and PSORTb (https://www.psort.org/psortb/). The results obtained are shown in Table S2. As for the distribution of *phoU* (*A1708*) in *Synechococcus* sp. PCC 7002. An alignment was made on Tara Ocean (http://tara-oceans.mio.osupytheas.fr/Ocean-gene-atlas/). The expectation threshold was set to 1E-8. The detailed operation process was carried out as described by Vernette et al. (Villar et al. 2018; Vernette et al. 2022).

## Results and discussion

### Transcriptional changes in *Synechococcus* sp. PCC 7002 under P starvation and P recovery conditions

**Fig. 1:**
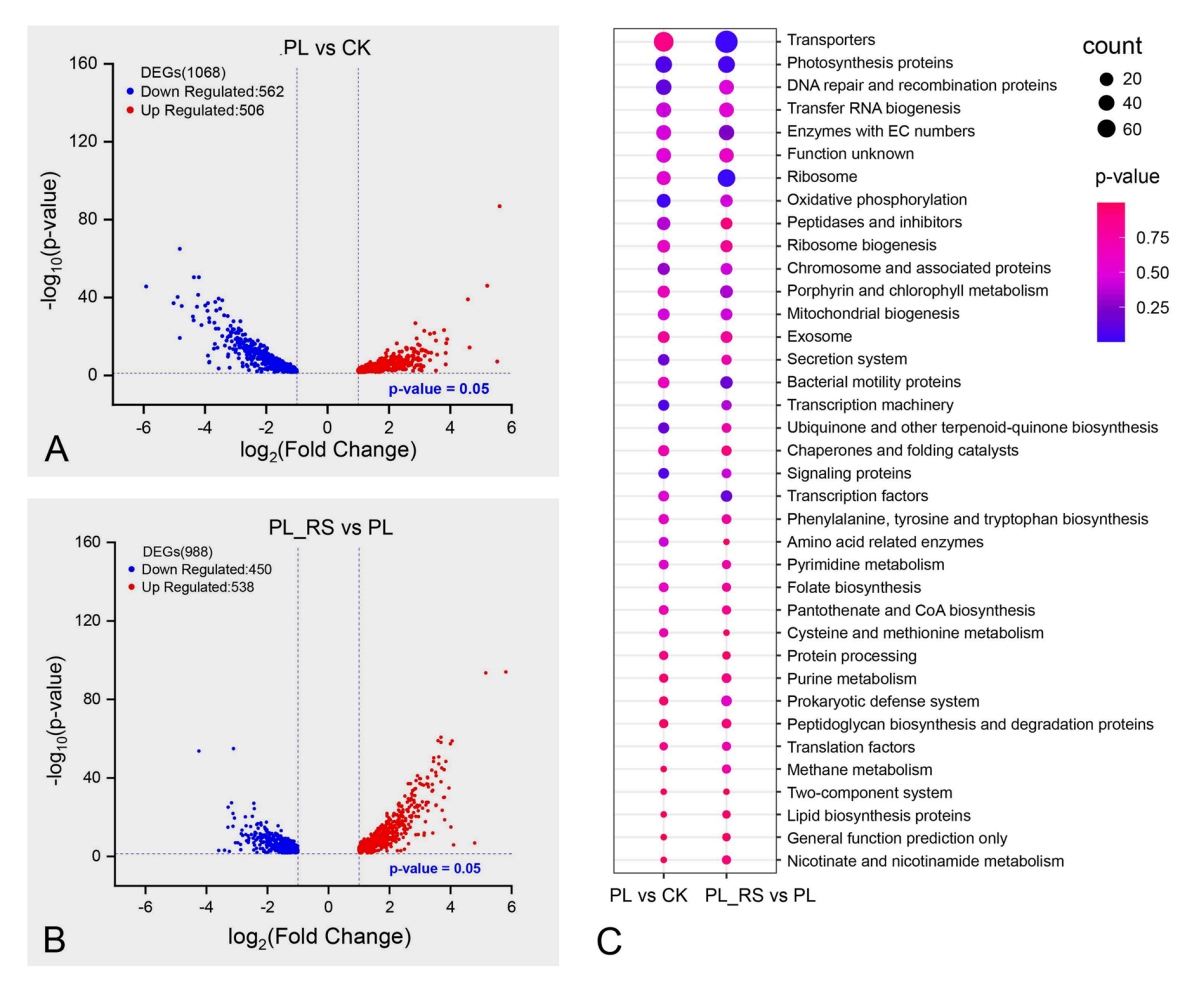
Analysis of up-regulated and down-regulated DEGs among different treatment groups. **A** and **B** Volcano maps of DEGs. **C** KEGG enrichment analysis during P deficiency and P recovery. The size of the dot depends on the number of genes enriched in the pathway, and the color of the dot indicates the significance of pathway enrichment. Where CK represents the normal culture, PL represents the P-deficient treatment, and PL_RS represents the P recovery treatment group

To explore the molecular mechanism of *Synechococcus* sp. PCC 7002 to the fluctuating P concentrations, we carried out transcriptome sequencing under three different conditions: standard P concentration (CK, 370 µmol/L), P deprivation for 4 days (PL, 0 µmol/L), and P recovery after 10 days of P deprivation (PL_RS, adding 370 µmol/L again). The DEGs were calculated using the DESeq2 R package based on the count matrix of different comparison groups. Although the up-regulated and down-regulated DEGs only showed the relatively gene expression levels between different comparison groups, the changes of the gene expression patterns also could offer valid information among different treatments. Our results showed that there were 1068 DEGs under P starvation conditions (PL vs CK), including 506 up-regulated genes and 562 down-regulated genes (Fig. 1A). A total of 988 DEGs were identified in PL_RS_vs_PL group, including 538 up-regulated genes and 450 down-regulated genes (Fig. 1B). These results indicated that *Synechococcus* sp. PCC 7002 can adapt to P starvation by down-regulating the expression of metabolism related genes that require P participation. It has been reported that open-ocean cyanobacterium *Synechococcus* sp. WH8102 exhibits a down-regulation of more genes after P deficiency (Tetu et al. 2009). Nonetheless, the opposite result was observed in the freshwater cyanobacterium *Raphidiopsis raciborskii* where more genes were up-regulated under P deficiency (Shi et al. 2022). These results suggest that different cyanobacteria may use alternative adaptation strategies to cope with changing P concentrations in the environment, especially between freshwater cyanobacteria and marine cyanobacteria.

The enriched KEGG pathways of the DEGs identified in PL_vs_CK comparison were the same as those in PL_RS_vs_PL comparison (Fig. 1C); however, the pattern of gene expression might be inverted. For example, most of the DEGs involved in photosynthesis, ribosome, and bacterial motility were down-regulated after P starvation whereas most of the DEGs involved in transporter, DNA repair and recombination, oxidative phosphorylation, tRNA synthesis, peptidase, porphyrin, and chlorophyll metabolism were up-regulated (Fig. S3A). In contrast, we found that most of the DEGs in transporter, porphyrin and chlorophyll metabolism, tRNA synthesis, and peptidase were down-regulated, while most DEGs involved in ribosome, photosynthesis, and bacterial motility were up-regulated in PL_RS vs PL group (Fig. S3B). It suggests that the metabolism state of the cyanobacterium cells will restore with the recovery of P concentration. It was also found in *Nostoc* sp. PCC 7118 and *Trichodesmium erythraeum* IMS101 that P supplementation after P starvation reversed the changes under cellular P starvation (Solovchenko et al. 2020; Frischkorn et al. 2019).

### Phosphorus deficiency has a great influence on photosynthesis and protein synthesis

**Fig. 2:**
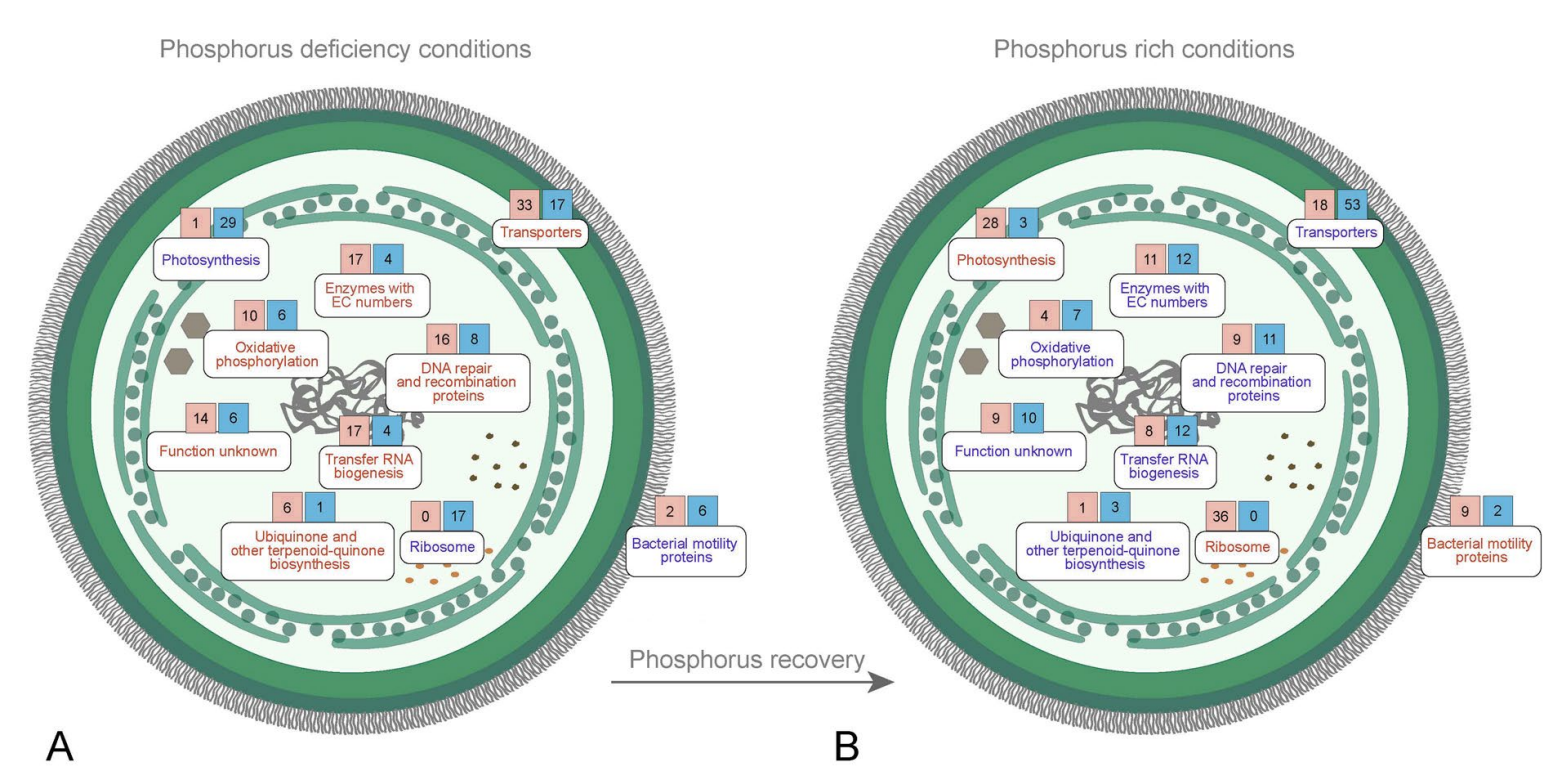
Effect of P deficiency and P recovery on metabolic pathways in *Synechococcus* sp. PCC 7002. **A** KEGG pathways under P deficiency treatment conditions. **B** KEGG pathways under P-rich treatment conditions after P deficiency. The red font represents the up-regulation of this metabolic pathway, the blue represents the down-regulation of this metabolic pathway, the number in the red square represents the number of up-regulated genes in the pathway in which it is located, and the blue square represents the number of down-regulated genes in the pathway in which it is located

Based on the results of the KEGG pathway enrichment analysis, we found that the pathways up-regulated during P starvation, such as photosynthetic, ribosomal, and motility proteins, were usually down-regulated after P recovery and those down-regulated pathways were then up-regulated after P recovery (Fig. 2), suggesting that these pathways are closely associated with P concentration changes. Photosynthesis is the fundamental pathways that focus on carbon fixation and energy metabolism. The inhibition of photosynthesis would severely influence the cell growth of cyanobacteria. In the PL vs CK group, the total count of DEGs implicated in photosynthetic proteins was 30, with down-regulation seen in 29 of them (Fig. 2). A comparable circumstance has been documented in *Microcystis* and *Trichodesmium*, illustrating that deficiencies in P may cause photosynthetic inhibition in many cyanobacteria. (Harke and Gobler 2013; Frischkorn et al. 2019).

Ribosomes are altered by P deprivation in addition to photosynthesis. The number of DEGs involved in ribosomes reached 17 in PL_vs_CK and 31 in PL_RS_vs_PL group, which were down-regulated and up-regulated, respectively (Fig. 2). P is mainly involved in nucleic acid synthesis and energy metabolism in cells. Ribosomal RNA (rRNA) is the most abundant RNA in cells, and P is an important component of rRNA. Therefore, intracellular rRNA will be reduced due to the lack of P. This will directly affect the synthesis of proteins in the cells, thus inhibiting the growth of strains. In addition, some cyanobacteria change the composition of intracellular metabolites to adapt to long-term P stress. For example, *Prochlorococcus* MED4 reduces its composition of P-containing compounds and increases the proportion of some essential metabolic proteins in a long-term P deficiency environment (Casey et al. 2016).

### Cells undergone P starvation and P recovery were metabolically more active than those in continuous P-rich culture

**Fig. 3:**
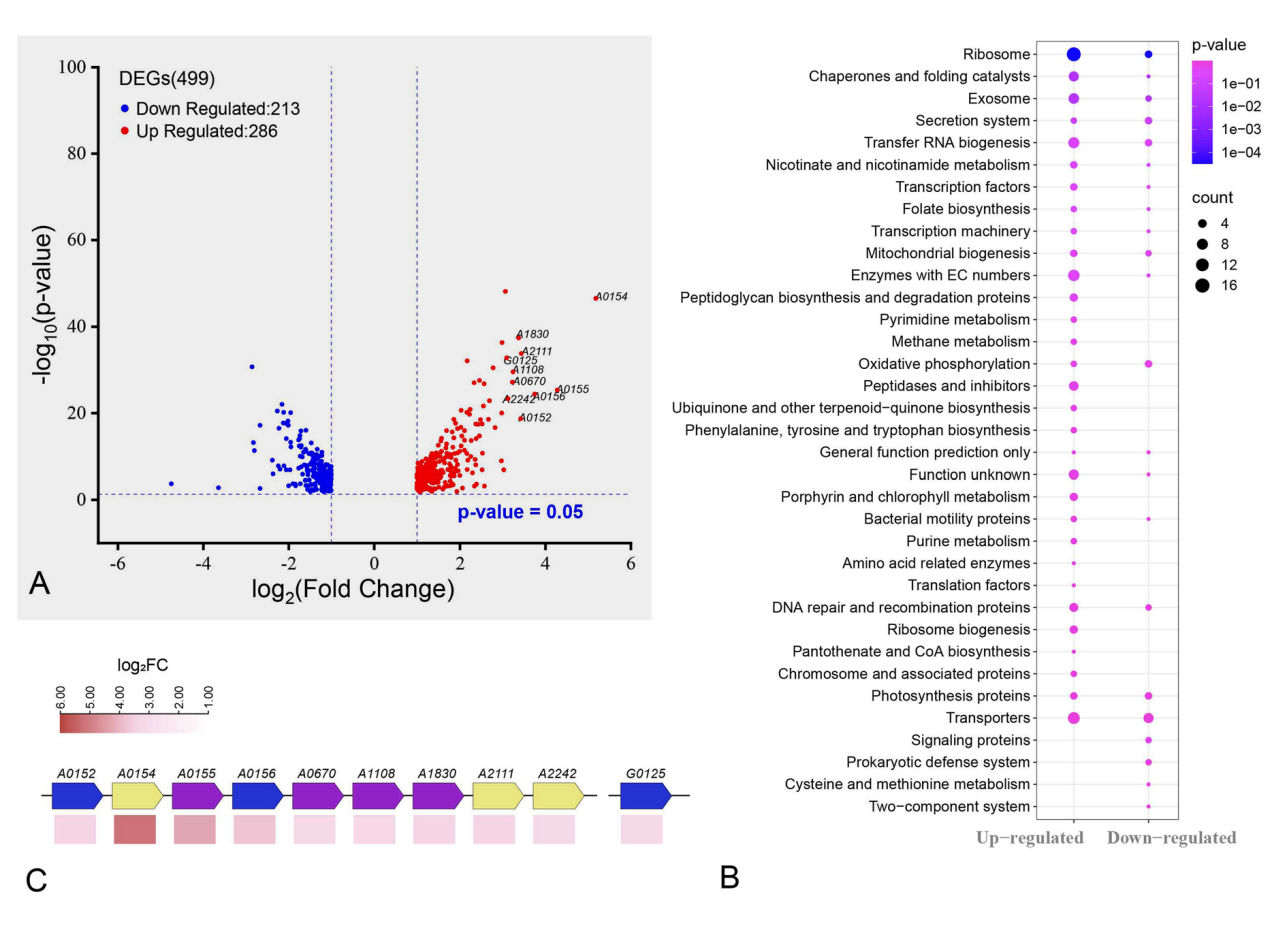
Analysis of DEGs between PL_RS group and CK treatment group. **A** Volcano map of DEGs. **B** KEGG enrichment analysis of DEGs between PL_RS group and CK treatment group. The size of the dot depends on the number of genes enriched in the pathway, and the color of the dot indicates the significance of pathway enrichment. **C** The distribution and expression of the ten genes with the highest up-regulated expression multiple in **A** on the genome. Wherein purple represents the enzyme encoded by the gene, blue represents the unknown function of the gene, and yellow represents that the gene encodes other proteins

There were 499 DEGs in PL_RS group compared with CK group, including 286 up-regulated genes and 213 down-regulated genes (Fig. 3A). Among 286 up-regulated genes, the ten most up-regulated genes include four genes encoding the synthesis of enzyme (*A0155*, *A0670*, *A1108*, *A1830*), three genes encoding ATP-binding protein (*A0154*), transcription initiation factor SinH (*A2111*), and ABC-2 transporter (*A2242*), respectively, and three function-unknown genes (Fig. 3C). The results of KEGG enrichment analysis in the PL_RS_vs_CK group indicated that the quantity of up-regulated genes in 27 KEGG pathways exceeded that of down-regulated genes (35 in total). It suggests that the vast majority of pathways are activated in the biological process in which they are situated. The three pathways in which the greatest number of genes was up-regulated were “Ribosome”, “Enzymes with EC numbers”, and “Transporters”. They are involved in protein synthesis, enzymatic reactions, and material transport which were closely related to cell metabolism. These results indicated that the substance synthesis of cyanobacterial cells undergoing P starvation and subsequent P recovery will be more active compared to cyanobacterial cells that were under continuous P-rich conditions.

Cyanobacteria have good adaptability to changing P concentration. Previous studies have shown that P deprivation can enhance the luxury uptake capacity of cyanobacterial cells during subsequent P recovery (Li et al. 2021; Wu et al. 2012). Cyanobacterial cells show changes in P uptake, polyphosphate turnover, and cell ultrastructure after the refeeding of P, which allows them to fully mobilize the uptake and utilization of P sources and prepare them to adapt to sudden changes in external P concentrations (Solovchenko et al. 2020). In our study, many metabolic pathways were up-regulated after P recovery compared to cyanobacteria that were kept in continuous P-replete conditions (Fig. 3B). Research has demonstrated that P-refeeding after P-starvation can increase the content of nutrients, such as PHB, PolyP, and cyanophycin granules in cyanobacterial cells compared with cells before starvation (Solovchenko et al. 2020). It suggests that cells need to uptake more P and other nutrients to restore the intracellular balance during P recovery. That is why, there were more up-regulation genes related to metabolic pathways in the P recovery treatment group.

### Genes related to P deficiency adaptation were screened and identified in *Synechococcus* sp. PCC 7002

Based on the transcriptome annotation data, 54 genes were selected for further investigation including phosphate transport, organophosphate utilization, indirect P metabolism, nutrient elements, and some genes with unknown functions (Table S1). The significant changes in transcription levels of these genes during P starvation (PL group) or P recovery (PL_RS group) indicated the potential roles of these genes in adaptation to changes in P concentrations. Furthermore, the selection of DEGs was guided by the reported P-deficient transcriptome of *Synechococcus* sp. PCC 7002 to avoid ignore crucial genes involved in the initial phase of P deficiency. To further reveal the accurate roles of these genes on cyanobacteria response to fluctuating P concentrations, gene knockout approach was used to obtain 44 mutant strains (Fig. S1). Four of these mutants could not be knocked out completely, and only knock-down mutants were obtained, namely Mut-A0549-50, Mut-A0556, Mut-A1895, and Mut-A2284-86. Genes that could not be knocked out completely may be crucial to survival.

The experimental results of P-limited culture of the 44 mutants showed that genes highly affected by P concentration at the transcriptional level did not necessarily show P-deficient sensitive phenotypes in their corresponding mutants. Finally, a total of 8 mutant strains were found with obvious growth differences from the wild type (WT). Their growth rates and the gene information are shown in Table 1. Five mutants (Mut-A0076, Mut-A0549-50, Mut-A1094, Mut-A1320, and Mut-A1895) were found to be sensitive to P-limited condition, with a significantly reduced growth rate compared to the WT. The six genes (*A0076*, *A0549*, *A0550*, *A1094*, *A1320*, and *A1895*) involved in these five mutants may play important roles in low-P adaptation, and their deletion leads to growth defects under P limitation. Also, the other three mutants (Mut-A0079, Mut-A0340, and Mut-A2284-86) displayed better growth under P-limited conditions when compared to the WT. The products of these genes (*A0079*, *A0340*, *A2284*, *A2285*, and *A2286*) may affect the adaptation of *Synechococcus* sp. PCC 7002 to P deficiency, but the specific mechanism still needs to be further studied. Furthermore, these genes were analyzed under P starvation and P recovery conditions at the transcriptional level and predicted for subcellular localization (Fig. S2 and Table S2). The results showed that *A0076*, *A0079*, *A0549*, *A0550*, *A1094*, and *A1895* are cytoplasmic proteins and the encoding products of the genes *A0340*, *A1320*, *A2285*, and *A2286* are membrane proteins. The protein encoded by *A2284* is located in the periplasmic space. The transcription levels of these 11 genes were significantly different at 24 h of P starvation, but half of the genes were not significantly different at 4 days of P starvation when compared to the control (CK) (Table S1 and Fig. S2). These results suggested that many low-P adaptation genes play a role in the early stage of P starvation, and their expression could be restored to the initial state after P recovery. Eleven genes could be divided into three categories based on gene annotation: metal-ion transporters, enzyme proteins, and phosphate transporters. The functions of genes that Mut-A0076 and Mut-A0079 knockouts were unknown, so they were not classified. The other six mutants were further analyzed in the following study.

**Table 1:** Growth rate ratios of *Synechococcus* sp. PCC 7002 between 8 mutant strains and WT cultured under P-deficient conditions and P standard conditions

Mutant strain	Knockout genes	Predicted gene function	Growth rate ratio (mutants/WT, standard P)	Growth rate ratio (mutants/WT, P deficiency)
Mut-A0076	*A0076*	Conserved hypothetical protein	98.18% ± 0.85%	85.84% ± 0.83%**
Mut-A0549-50	*A0549*	Metal-dependent phosphoesterases (PHP family), putative	98.61% ± 0.99%	91.02% ± 1.60%**
	*A0550*	Amidophosphoribosyltransferase		
Mut-A1094	*A1094*	Putative succinate dehydrogenase iron-sulfur protein	97.46% ± 0.14%	90.43% ± 2.40%**
Mut-A1320	*A1320*	Chromate transporter	98.84% ± 2.61%	63.99% ± 2.07%**
Mut-A1895	*A1895*	Phosphate import ATP-binding protein	97.35% ± 2.51%	90.85% ± 1.61%**
Mut-A0079	*A0079*	Conserved hypothetical protein	98.72% ± 2.37%	107.2% ± 1.37%*
Mut-A0340	*A0340*	HupE/UreJ family protein	101.87% ± 0.97%	119.47% ± 1.17%**
Mut-A2284-86	*A2284*	Phosphate transport system substrate-binding protein	98.19% ± 4.27%	107.81% ± 0.93%*
	*A2285*	Phosphate ABC transporter permease subunit PstC		
	*A2286*	Phosphate ABC transporter, permease protein		

^a^The statistical analysis method used is the independent-sample *t* test. **significance at *p* < 0.01; *significance at *p* < 0.05. The ± values indicate standard deviations from three biological replicates

### Metal-ion transporters and certain metabolic processes may play important roles in adapting to P starvation

**Fig. 4:**
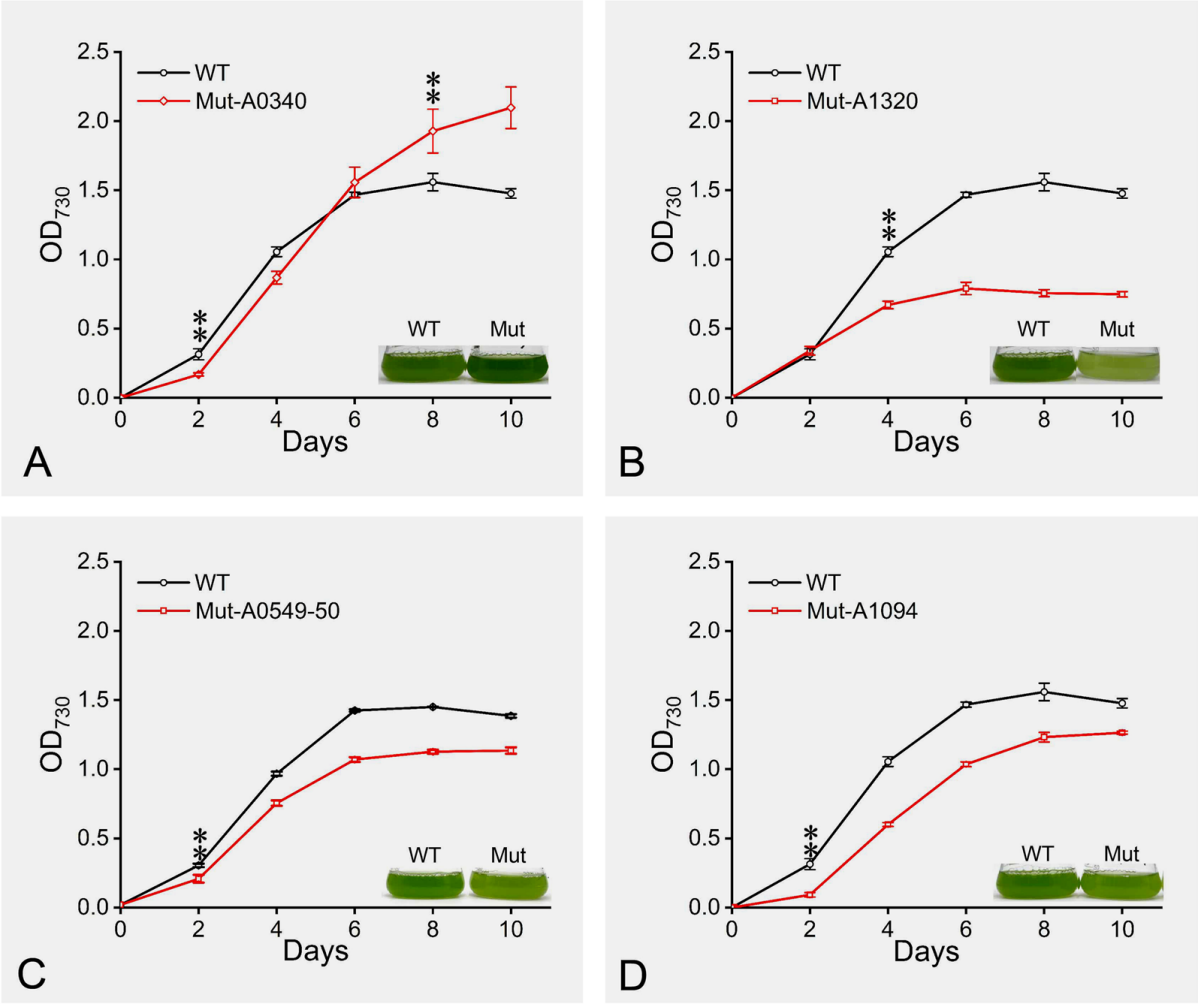
Mutant growth. **A–D** The growth curve and picture of strains in phosphor-limited culture. **Represents a very significant difference (*p* < 0.01). Error bars indicate the standard deviation from three biological replicates

The genes involved in Mut-A0340 and Mut-A1320 mutants encode two metal-ion transporters. *A0340* encodes UreJ protein, a common metal-ion transporter in various bacteria (Baginsky et al. 2004), which is primarily involved in the transport of nickel ions. *A1320* encodes the chromate transporter (ChrA), which is involved in the efflux of chromate ions (Aguilera et al. 2004; Rafael et al. 2006). *A0340* was down-regulated during 4 days of P deprivation and up-regulated after P was resupplied (Fig. S2). It suggests that *A0340* may be detrimental to adapt to P deficiency. The results of physiological experiments showed that the growth of the Mut-A0340 mutant strain was better than the WT under P deficiency (Fig. 4A). This phenomenon was consistent with our transcriptional changes after 4 days of P deficiency, revealing its possible negative effect on cellular adaptation to P deficiency. The expression of *A1320* was up-regulated after P deprivation and down-regulated after P recovery (Fig. S2). In addition, Mut-A1320 exhibited a phosphate-deficient sensitive phenotype (Fig. 4B). These findings suggested that *A1320* may play an important role in the adaptation to P deficiency.

Mut-A1094 and Mut-A0549-50 showed significantly inhibited growth under P-limited culture compared to the WT (Fig. 4C and D). The enzyme succinate dehydrogenase iron–sulfur protein subunit (SdhB) encoded by *A1094* is involved in the biological process of succinate to fumaric acid in the TCA cycle, which is linked to the synthesis of intracellular ATP. The growth of Mut-A1094 may be inhibited by the deletion of *A1094*, which could limit the cell energy supply in the event of a P deficit. *A0549* encodes metal-dependent phosphoesterases that catalyzes the hydrolysis of phosphate ester bonds. PurF (*A0550*) is an amidophosphoribosyltransferase that is involved in the synthesis of PRA (5-phospho-D-ribosylamine), the first step in purine biosynthesis (Malkowski et al. 2020). We hypothesized that the P-deficient sensitive phenotype of Mut-A0549-50 may result from the loss of function of *A0549* based on the functions of these two genes.

### Inorganic phosphate transport system is vital for *Synechococcus* sp. PCC 7002 to adaptation to P starvation

The genes knocked down in Mut-A1895 and Mut-A2284-86 (*pstSCAB*) were involved in inorganic phosphate transport. The inorganic phosphate transport system includes the substrate-binding protein PstS, phosphate ABC transporter PstCA, and phosphate transport system ATP-binding protein PstB. These four genes (*pstSCAB*) were significantly up-regulated after 24 h of P starvation. Furthermore, *pstS* and *pstB* remained up-regulated after 4 days of P deprivation (Table S1 and Fig. S2). These results indicate that *pstB* and *pstS* may be critical in the adaptation to P deficiency. Similarly, previous research also found the key function of these two genes response to P deficiency in other cyanobacterial species (Tetu et al. 2009). However, there was no significant difference in these four genes after P recovery (Fig. S2), demonstrating that Pst system may be activated only under P insufficient conditions.

**Fig. 5:**
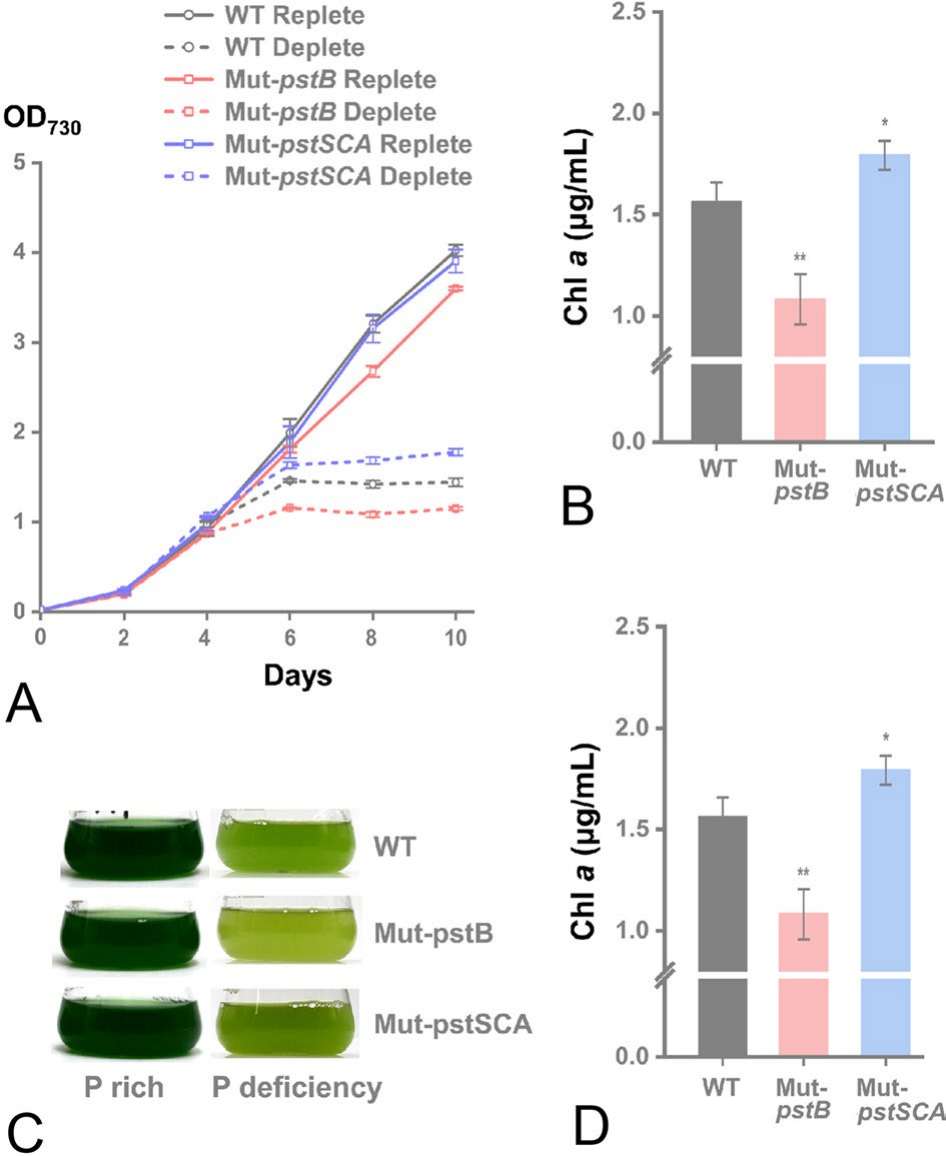
Physiological phenotypes of phosphate transport-related mutant strains and WT in *Synechococcus* sp. PCC 7002. **A** The growth curve of the mutant strain in phosphor-limited culture. **B** Chl *a* content of mutant and WT in P-rich and P-deficient conditions. **C** Picture of mutant and WT in P-rich and P-deficient conditions. **D** *Fv/Fm* (ratio of variable to maximum fluorescence) of mutant and WT in P-rich and P-deficient conditions. **Represents a very significant difference (*p* < 0.01). Error bars indicate the standard deviation from three biological replicates

In our study, Mut-A1895 and Mut-A2284-86 were knock-down strains, and these genes could not be completely knocked out. The probable reason is that *Synechococcus* sp. PCC 7002 inhabits a coastal environment with variable P concentration, where the Pst system is crucial for survival. Localized in the Pst system, the *pstS* gene has been expressed in cyanobacteria under P starvation (Pereira et al. 2019). The growth of mutant strain Mut-A1895 was significantly lower than that of WT from the second day of P limitation culture, while the Mut-A2284-86 was significantly higher than that of the WT from the fourth day (Fig. 5). Functional defects in PstB affect ATP hydrolysis which may be responsible for the growth defects. A total of three proteins in Mut-A2284-86 (PstS, PstC, and PstA) were functional defects. In the periplasmic space, PstS binds P with high affinity, and it has been suggested that it may serve as a major sensor for external phosphates (Wanner 1993). We speculate that PstS may play a role in negatively regulating adaptation during P starvation. However, we cannot exclude the effect of PstC and PstA, and more specific P transport mechanisms must be explored.

### Possible regulatory strategies in the adaptation of *Synechococcus* sp. PCC 7002 to fluctuating P concentrations

**Fig. 6:**
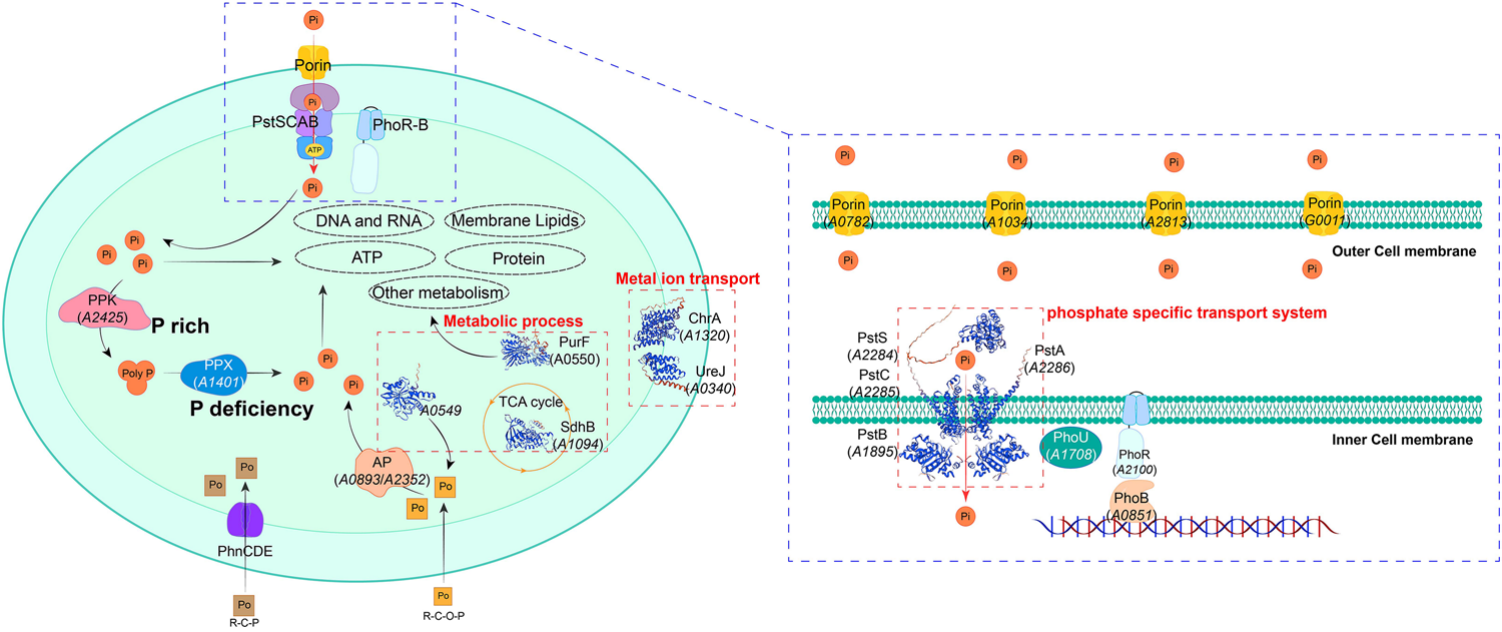
Pattern of possible P uptake and transport in *Synechococcus* sp. PCC 7002. Pi stands for inorganic phosphate and Po stands for organophosphorus. Among them, Po is divided into phosphonate (R–C–P) and phosphate (R–C–O–P). The important genes identified in this study were predicted by tertiary structure modeling and subcellular localization. The genes identified in the red dotted box may play an important role in the process of phosphorus concentration adaptation

**Fig. 7:**
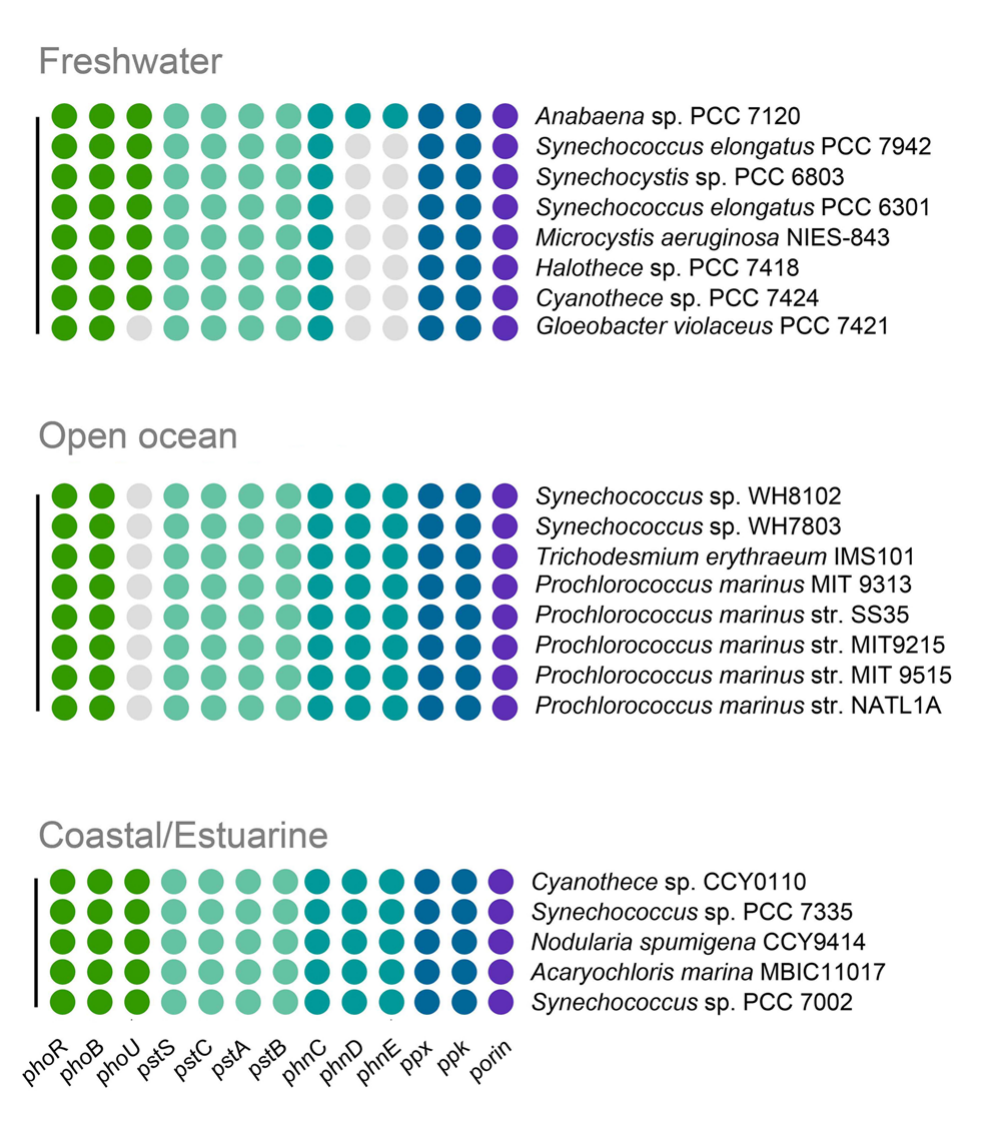
Diversity analysis of phosphate uptake and transport pathways in different cyanobacteria from different habitats. Distribution of genes related to phosphorus uptake and utilization in different cyanobacteria. The gray area represents the absence of this gene in this species

According to the information reported in the existing literature and the results of this study, we summarized and analyzed P acquisition strategies of *Synechococcus* sp. PCC 7002 and drew a model diagram (Fig. 7). Inorganic phosphate from the environment enters the periplasmic space via four porins (*A0782*, *A1034*, *A2813*, and *G0011*) on the outer membrane and enters the cytoplasm via the Pst system on the inner membrane. The function of Pst system is regulated by the two-component PHO regulator PhoR-B. This system has been found in many cyanobacterial species detected in marine ecosystems as well as in freshwater ecosystems, and their function may be similar to those in heterotrophic bacteria (Harke and Gobler 2013; Peterson et al. 2005; Scanlan et al. 2009; Sinha et al. 2014). In bacteria, when external P is insufficient, PhoR is activated by autophosphorylation which further induces the phosphorylation of PhoB. Subsequently, this activates the expression of PHO regulator genes and then enhances P uptake and assimilation (Srikumar et al. 2015; Jarvik et al. 2010). Phosphate that enters the cell can be used to synthesize substances such as nucleic acids, proteins, membrane lipids, and ATP (Fig. 6). In the presence of sufficient phosphate, the remaining phosphate forms polyphosphate (polyP) in response to PPK. Once phosphate is scarce, the polyP was hydrolyzed in response to PPX and is then used for survival.

In addition to inorganic phosphorus (Pi), cyanobacteria can use organic phosphorus (Po). Dissolved organophosphate in water mainly includes phosphate monoesters and phosphonates (Clark et al. 1998; Kolowith et al. 2001). The Phn transport system, including PhnC (*A0713, G0141*), PhnD (*A0336, G0143*), and PhnE (*A0759, G0142*), is present in the *Synechococcus* sp. PCC 7002 cell membrane which is related to the uptake and transport of phosphonate (Fig. 6). This system has been found in many marine cyanobacteria (Dyhrman et al. 2006). Alkaline phosphatase (AP) is required for the decomposition and utilization of phosphate monoesters. There are two putative genes encoding AP (*A0893, A2352*) in *Synechococcus* sp. PCC 7002. Extracellular alkaline phosphatases can hydrolyze Po into Pi around cells, a suitable form that can be absorbed and utilized. In the case of the P starvation, *Synechococcus* sp. PCC 7002 can mobilize other forms of P sources to use, thus adapting to the changes in the P concentration in the environment.

Proteins that do not participate directly in the P acquisition pathways may also play important regulatory roles in adaptation to changes in P concentration, especially those in important metabolic processes, such as *A0549*, *A0550*, and *A1094*. They are involved in phosphate ester hydrolysis, purine synthesis, and TCA cycle, respectively. Cyanobacteria cells may be regulated by these metabolic processes under the condition of P deficiency. In addition, some non-phosphate transporters are important in adapting to the change of P concentration, such as two metal-ion transporters encoded by *A0340* and *A1320*. These results suggested that some important metabolic processes and substance transport systems played important roles for *Synechococcus* sp. PCC 7002 to adapt to the change of P concentration. More importantly, *Synechococcus* sp. PCC 7002 has complex P regulation mechanisms and abundant P uptake pathways to cope with the drastic changes in P concentration.

### Coastal cyanobacteria have more complex regulatory mechanisms for P uptake than those open-ocean species

*Synechococcus* sp. PCC 7002 is a typical cyanobacterial strain living in coastal or estuarine environments where P concentration is frequently fluctuating due to the influx of terrestrial fresh water and nutrients, as well as tidal influences (Chanvalon et al. 2016). Thus, sophisticated P acquisition strategies may be necessary to adapt to such environments for these kinds of phytoplankton species. However, whether cyanobacteria growing in different habitats have similar P uptake and regulation mechanisms is need to be further investigated. Therefore, the distribution and comparison of genes related to P uptake and utilization of some typical cyanobacteria from freshwater, ocean, and coastal areas were analyzed (Fig. 7).

Hypoxic and iron-rich conditions have dominated the ocean interior in most of the earth history period (Planavsky et al. 2011; Sperling et al. 2015). Bioavailable P is bound by Fe^2+^, leading to long-term P deficiency (Derry 2015). P uptake is particularly important in this context. Thus, the Pst system, passive diffusion pathways (such as *porin*), and polyphosphate metabolic pathways (such as *ppk* and *ppx*) are indispensable in most cyanobacteria species (Fig. 7). Besides these genes, our results showed that the coastal/estuarine cyanobacteria species almost possessed all the related genes taking part in P uptake pathways and P regulation mechanisms which would make them profitable in the regions with sharply P concentration fluctuating conditions.

However, open-ocean lacks multiple exogenous P inputs that can be found in coastal/estuarine and freshwater environments. P concentration remains relatively low but stable in open-ocean regions (Thingstad et al. 2005). Therefore, the complex P regulation mechanisms may not be essential for these cyanobacteria species habitat in open-ocean. The results of the homology comparison showed that *phoRB* was found in all cyanobacteria, while *phoU* cannot be found in open-ocean cyanobacteria (Fig. 7). In addition, analysis of Tara Ocean data showed that *phoU* were mainly distributed in coast or offshore which were not found in the open-ocean regions (Fig S4). Moreover, *phoU* was considered as a putative negative regulator to regulate the cellar P homeostasis (Surachet et al. 2009). Thus, *phoU* of the open-ocean cyanobacteria species might have been abandoned during the long-term evolution. In addition, to gain competitive advantage in P-deficient environment, the open-ocean cyanobacteria species reserved the alternative P transporter, such as PhnDE, which were reported to be affinity to various forms of P sources in marine cyanobacteria (Shah et al. 2023). These results indicated that divers P uptake pathways would be beneficial for the open-ocean cyanobacteria to survive in relatively low but stable P environment. *PhnDE* is commonly found in open-ocean cyanobacteria but is absent in most freshwater cyanobacteria (Fig. 7). Instead, the freshwater cyanobacteria as well as coastal/estuarine cyanobacteria species reserved the *phoU* gene to regulate the cellar P homeostasis under elevated P concentrations with the land-based P inputs or groundwater discharges. Notably, cyanobacteria such as *Microcystis* in freshwater environments have distinct P utilization mechanisms. The inorganic P affinity and P uptake rate of *Microcystis* were discovered to be lower to those of oligotrophic water-dominating cyanobacteria (Mulder et al. Mulder and Hendriks 2014; Vadstein 2000). However, the strong P storage capacity of *Microcystis* and the allelopathy that can be triggered by P source help them gain a competitive advantage in the eutrophic freshwater environment (Marinho et al. 2013; Zhang et al. 2021).

It has been reported that most modern cyanobacterial species may have emerged after the Snowball Earth event, flowing from freshwater to the ocean (Hoffman et al. 2017). Coastal/Estuarine are between land and sea, which are environmentally sensitive zones causing frequent environmental changes for the species (Korsman et al. 2014; Abbate et al. 2017). To cope with the drastic changes in P concentration, diverse P transport pathways could help such cyanobacteria species in the region to take up enough P when P concentration is insufficient. On the other hand, more complex phosphate regulation system mechanism is beneficial for cyanobacteria to maintain the balance of P uptake. It is similar to our previous findings on iron uptake strategies that the iron acquisition pathways of coastal cyanobacteria are more diverse than those of open-ocean cyanobacteria (Yong et al. 2023). In conclusion, we suggest that cyanobacteria may have more sophisticated P acquisition strategies and regulatory mechanisms in environments with highly fluctuating P concentrations.

### Supplementary Information

Below is the link to the electronic supplementary material.

Supplementary file1 (PDF 1024 KB)

## Data Availability

Most data generated or analyzed during this study are included in this published article and its supplementary information files. The transcriptome data can be accessed in Genome Sequence Archive (GSA: CRA014148, https://ngdc.cncb.ac.cn/gsa).
