# Supplementary material for: Molecular mechanism of a coastal cyanobacterium Synechococcus sp. PCC 7002 adapting to changing phosphate concentrations
Source: Mar Life Sci Technol. 2024 Jul 22;6(3):562–75. doi: 10.1007/s42995-024-00244-y (PMC11358556; doi:10.1007/s42995-024-00244-y)
Supplement: Supplementary file 1 — Supplementary file1 (PDF 1024 KB) [file 42995_2024_244_MOESM1_ESM.pdf]

Supplementary information

Molecular mechanism of a coastal cyanobacterium *Synechococcus* sp. PCC 7002 adapting to changing phosphate concentrations

Qiao-Wei Sun [a, b], Yu Gao [c], Jordan Wang [d], Feixue Fu [d], Cheng-Wen Yong [a], Shuang-Qing Li [a], Hai-Long Huang [a, b], Wei-Zhong Chen [a], Xin-Wei Wang[a, b] * Hai-Bo Jiang [a, b] *

[a] School of Marine Sciences, Ningbo University, Ningbo, Zhejiang 315211, China

[b] Southern Marine Science and Engineering Guangdong Laboratory (Zhuhai), Zhuhai, Guangdong 519080, China

[c] School of Life Sciences, Central China Normal University, Wuhan 430079, People's Republic of China

[d] Department of Biological Sciences, University of Southern California, Los Angeles, CA 90089, U.S.A.

* Corresponding authors:

X.W. Wang (wangxinwei@nbu.edu.cn) and H.B. Jiang (jianghaibo@nbu.edu.cn)

Qiao-Wei Sun and Yu Gao contributed equally to this study.

Table S1 Function and transcriptional changes of genes related to low phosphorus adaptation in *Synechococcus* sp. PCC 7002.

Classification	Gene code	Predicted gene function	a	b	c	d	Gene localization
Utilization of organophosph-orus	*A0148*	two-component hybrid sensor and regulator; PAS domain S-box protein	2.05	ND	ND	ND	pAQ2:149816-153046
	A0149	glycosyl transferase, group 1 family protein	3.99	ND	ND	ND	pAQ2:153399-154799
	A0150	bifunctional metallophosphatase/5'-nucleotidase	5.16	ND	-1.11	-1.31	pAQ2:154890-156881
	A0539	putative kinase	1.67	ND	ND	ND	pAQ2:569794 - 570318
	A0549	metal-dependent phosphoesterases (PHP family), putative	-0.97	ND	ND	ND	pAQ2:576633 - 577325
	A0550	Amidophosphoribosyltransferase	-1.18	-1.72	2.03	ND	pAQ2:577569 - 579038
	A0893	phosphatase	7.18	4.58	-4.25	ND	pAQ2:924089 - 925924

	A2352	ferredoxin: protochlorophyllide reductase (ATP-dependent) iron-sulfur ATP-binding protein	6.16	ND	ND	ND	pAQ2:2430554 - 2432035
	G0067	ExeM/NucH family extracellular endonuclease	5.93	ND	-1.45	ND	pAQ7:68503 - 73563
	G0068	peptidylprolyl isomerase	5.03	-1.39	ND	-1.21	pAQ7:74140 - 74874
Transport of phosphate	*A1895*	Phosphate import ATP-binding protein	2.89	2.42	-2.09	ND	pAQ2:1979838 - 1980641
	A2284	phosphate transport system substrate-binding protein	5.13	1.73	-1.16	ND	pAQ2:2367149 - 2368213
	A2285	phosphate ABC transporter permease subunit PstC	3.49	-1.29	ND	-1.16	pAQ2:2368344 - 2369282
	A2286	phosphate ABC transporter, permease protein	3.07	-1.04	ND	ND	pAQ2:2369279 - 2370190
	G0069	type I secretion system permease/ATPase	3.65	-1.01	ND	ND	pAQ7:74934 - 77972
	G0070	HlyD family efflux transporter periplasmic adaptor subunit	3.41	-3.25	1.05	-2.13	pAQ7:78123 - 79721
	A0250	phosphoenolpyruvate synthase	2.32	ND	ND	ND	pAQ2:263501 - 266002
Indirect phosphorus metabolism	*A0667*	sll0787 family AIR synthase-like protein	1.33	2.16	-2.01	ND	pAQ2:697769 - 698746
	A0556	ATP-binding protein	1.48	-1.97	1.08	ND	pAQ2:582725 - 583189
	A1077	cyclic nucleotide-binding domain-containing protein	2.22	1.93	ND	ND	pAQ2:1107536 - 1107910
	A2697	glyceraldehyde-3-phosphate dehydrogenase, type I	2.64	1.26	-1.27	ND	pAQ2:2826736 - 2827779

	A2833	type I glyceraldehyde-3-phosphate dehydrogenase	2.21	ND	ND	ND	pAQ2:2956689 - 2957591
	A0340	HupE/UreJ family protein	1.64	-1.07	1.79	ND	pAQ2:356688 - 357293
Nutrient element related	*A0424*	aldo/keto reductase	-1.64	1.34	-2.01	ND	pAQ2:444802 - 445926
	A0685	ATP-dependent zinc protease	1.59	2.37	-2.38	ND	pAQ2:714052 - 714510
	A1094	putative succinate dehydrogenase iron-sulfur protein	1.66	ND	1.29	ND	pAQ2:1123904 - 1124905
	A1232	helix-turn-helix domain-containing protein	2.88	-1.06	1.27	ND	pAQ2:1275401 - 1275997
	A2347	"light-independent protochlorophyllide reductase, iron-sulfur ATP-binding protein"	1.79	ND	1.52	ND	pAQ2:1275401 - 1275997
	A2493	phosphate-starvation-inducible PsiE family protein	1.59	ND	-1.52	ND	pAQ2:2598334 - 2598663
	A0873	pentapeptide repeat-containing protein	1.81	-2.18	2.21	ND	pAQ2:903161 - 904024
	A0922	conserved hypothetical protein (glyco-hydrolase domain, family 57)	1.68	ND	ND	ND	pAQ2:953618 - 955084
Other Functions	*A0960*	GFA family protein	-1.60	ND	ND	ND	pAQ2:990060 - 990461
	A1034	carbohydrate porin	3.39	1.12	-1.13	ND	pAQ2:1070298 - 1072124
	A1204	alpha/beta hydrolase	1.93	-1.27	ND	ND	pAQ2:1249378 - 1250001
	A1320	chromate transporter	3.90	ND	-1.04	ND	pAQ2:1374890 - 1376029

	A1623	C-3',4' desaturase CrtD	-1.51	-1.07	1.22	ND	pAQ2:1703613 - 1705112
	A2472	ribonuclease HII	1.57	ND	ND	ND	pAQ2:2576400 - 2577002
	A2473	segregation and condensation protein B	1.60	1.56	ND	ND	pAQ2:2577051 - 2577545
	A2598	Mo-dependent nitrogenase	2.24	ND	ND	1.91	pAQ2:2721347 - 2724112
	A2599	response regulator	2.05	ND	ND	ND	pAQ2:2724159 - 2727203
	A2602	two-component response regulator	1.67	1.25	-1.54	ND	pAQ2:2728190 - 2729314
	A2687	response regulator	1.62	ND	-1.35	ND	pAQ2:2816530 - 2817567
Unknown function	*A0076*	conserved hypothetical protein	1.68	ND	ND	ND	pAQ2:82165 - 82413
	A0079	conserved hypothetical protein	2.62	ND	ND	ND	pAQ2:83336 - 83683
	A0479	conserved hypothetical protein	1.70	ND	ND	ND	pAQ2:510216 - 510848
	A0645	conserved hypothetical protein	1.77	ND	ND	ND	pAQ2:675502 - 676212
	A0709	conserved hypothetical protein	1.94	ND	ND	ND	pAQ2:741040 - 741534
	A0779	conserved hypothetical protein	1.63	-1.06	ND	ND	pAQ2:807703 - 808086
	A0892	hypothetical protein, partial	5.20	ND	-1.53	ND	pAQ2:923329 - 923955
	A1249	conserved hypothetical membrane protein	1.76	ND	ND	ND	pAQ2:1290109 -

							1290429
	A1339	conserved hypothetical protein	1.92	ND	ND	ND	pAQ2:1392621 - 1392923
	A1360	hypothetical protein	2.05	-1.76	ND	ND	pAQ2:1420100 - 1420588
	A1429	conserved hypothetical protein	1.88	ND	1.11	ND	pAQ2:1501544 - 1501750
	A2101	hypothetical protein	2.21	-1.08	1.64	ND	pAQ2:2177815 - 2178225
	F0050	hypothetical protein	1.67	ND	ND	ND	pAQ6:52530 - 52754
	F0106	hypothetical protein	1.67	ND	ND	-1.49	pAQ6:122765 - 123112

ND indicates non-differentially expressed genes. a: Transcription changes of phosphorus deficiency for 24 hours compared with phosphorus enrichment culture (the transcriptome data from the work of Ludwig and Bryant). b: Transcriptional changes in p-deficient treatment for 4 days compared with P rich cultures. c: Transcriptional changes in the P recovery treatment compared with the 4 days P deprivation treatment group. d: Transcriptional changes in P recovery treatment versus P rich culture.

Table S2 Subcellular localization prediction results

Genes	Gneg-mPLoc	CELLO	PSORTb	Transmembrane structure
A0076	Inner membrane. Cytoplasmic	Cytoplasmic	unknown	no
A0079	Extracellular	Cytoplasmic	unknown	no
A0340	Inner membrane.	Inner membrane.	Inner membrane.	yes
A0549	Cytoplasmic	Cytoplasmic Periplasmic	unknown	no
A0550	Cytoplasmic	Cytoplasmic	Cytoplasmic	no
A1094	Inner membrane.	Cytoplasmic Periplasmic	Inner membrane.	no
A1320	Inner membrane.	Inner membrane.	Inner membrane.	yes
A1895	Inner membrane.	Cytoplasmic Periplasmic	Inner membrane.	no
A2284	Periplasmic	Extracellular	Inner membrane.	no
A2285	Inner membrane.	Inner membrane.	Inner membrane.	yes
A2286	Inner membrane.	Inner membrane.	Inner membrane.	yes

Table S3 Species used in this study

Strain	Description	Source
Synechococcus sp. PCC 7002	Wild type	Laboratory preservation
Mut-A0076	Sp^r, *Synechococcus* 7002 mutant, missing genetic *A0076,* result of transformation with *A0076*::Ω	This study
Mut-A0079	Sp^r, *Synechococcus* 7002 mutant, missing genetic *A0079,* result of transformation with *A0079*::Ω	This study
Mut-A0148-50	Sp^r, *Synechococcus* 7002 mutant, missing genetic *A0148, A0149 and A0150,* result of transformation with *A0148-50*::Ω	This study
Mut-A0250	Sp^r, *Synechococcus* 7002 mutant, missing genetic *A0250,* result of transformation with *A0250*::Ω	This study
Mut-A0340	Sp^r, *Synechococcus* 7002 mutant, missing genetic *A0340,* result of transformation with *A0340*::Ω	This study
Mut-A0424	Sp^r, *Synechococcus* 7002 mutant, missing genetic *A0424,* result of transformation with *A0424*::Ω	This study
Mut-A0479	Sp^r, *Synechococcus* 7002 mutant, missing genetic *A0479,* result of transformation with *A0479*::Ω	This study
Mut-A0539	Sp^r, *Synechococcus* 7002 mutant, missing genetic *A0539,* result of transformation with *A0539*::Ω	This study
Mut-A0549-50	Sp^r, *Synechococcus* 7002 mutant, missing genetic *A0549* and *A0550,* result of transformation with *A0549-50*::Ω	This study
Mut-A0556	Sp^r, *Synechococcus* 7002 mutant, missing genetic *A0556,* result of transformation with *A0556*::Ω	This study
Mut-A0645	Sp^r, *Synechococcus* 7002 mutant, missing genetic *A0645,* result of transformation with *A0645*::Ω	This study
Mut-A0667	Sp^r, *Synechococcus* 7002 mutant, missing genetic *A0667,* result of transformation with *A0667*::Ω	This study
Mut-A0685	Sp^r, *Synechococcus* 7002 mutant, missing genetic *A0685,* result of transformation with	This study

	A0685::Ω	
Mut-A0709	Sp^r, *Synechococcus* 7002 mutant, missing genetic *A0709,* result of transformation with *A0709*::Ω	This study
Mut-A0779	Sp^r, *Synechococcus* 7002 mutant, missing genetic *A0779,* result of transformation with *A0779*::Ω	This study
Mut-A0873	Sp^r, *Synechococcus* 7002 mutant, missing genetic *A0873,* result of transformation with *A0873*::Ω	This study
Mut-A0892-93	Sp^r, *Synechococcus* 7002 mutant, missing genetic *A0892* and *A0893,* result of transformation with *A0892-93*::Ω	This study
Mut-A0922	Sp^r, *Synechococcus* 7002 mutant, missing genetic *A0922,* result of transformation with *A0922*::Ω	This study
Mut-A0960	Sp^r, *Synechococcus* 7002 mutant, missing genetic *A0960,* result of transformation with *A0960*::Ω	This study
Mut-A1077	Sp^r, *Synechococcus* 7002 mutant, missing genetic *A1077,* result of transformation with *A1077*::Ω	This study
Mut-A1094	Sp^r, *Synechococcus* 7002 mutant, missing genetic *A1094,* result of transformation with *A1094*::Ω	This study
Mut-A1204	Sp^r, *Synechococcus* 7002 mutant, missing genetic *A1204,* result of transformation with *A1204*::Ω	This study
Mut-A1232	Sp^r, *Synechococcus* 7002 mutant, missing genetic *A1232,* result of transformation with *A1232*::Ω	This study
Mut-A1249	Sp^r, *Synechococcus* 7002 mutant, missing genetic *A1249,* result of transformation with *A1249*::Ω	This study
Mut-A1320	Sp^r, *Synechococcus* 7002 mutant, missing genetic *A1320,* result of transformation with *A1320*::Ω	This study
Mut-A1339	Sp^r, *Synechococcus* 7002 mutant, missing genetic *A1339,* result of transformation with *A1339*::Ω	This study
Mut-A1360	Sp^r, *Synechococcus* 7002 mutant, missing genetic *A1360,* result of transformation with *A1360*::Ω	This study

Mut-A1429	Sp^r, *Synechococcus* 7002 mutant, missing genetic *A1429,* result of transformation with *A1429*::Ω	This study
Mut-A1623	Sp^r, *Synechococcus* 7002 mutant, missing genetic *A1623,* result of transformation with *A1623*::Ω	This study
Mut-A1895	Sp^r, *Synechococcus* 7002 mutant, missing genetic *A1895,* result of transformation with *A1895*::Ω	This study
Mut-A2101	Sp^r, *Synechococcus* 7002 mutant, missing genetic *A2101,* result of transformation with *A2101*::Ω	This study
Mut-A2284-86	Sp^r, *Synechococcus* 7002 mutant, missing genetic *A2284*, *A2285* and *A2286*, result of transformation with *A0549-50*::Ω	This study
Mut-A2347	Sp^r, *Synechococcus* 7002 mutant, missing genetic *A2347,* result of transformation with *A2347*::Ω	This study
Mut-A2352	Sp^r, *Synechococcus* 7002 mutant, missing genetic *A2352,* result of transformation with *A2352*::Ω	This study
Mut-A2472-73	Sp^r, *Synechococcus* 7002 mutant, missing genetic *A2472* and *A2473,* result of transformation with *A2472-73*::Ω	This study
Mut-A2598-99	Sp^r, *Synechococcus* 7002 mutant, missing genetic *A2598* and *A2599,* result of transformation with *A2598-99*::Ω	This study
Mut-A2602	Sp^r, *Synechococcus* 7002 mutant, missing genetic *A2602,* result of transformation with *A2602*::Ω	This study
Mut-A2687	Sp^r, *Synechococcus* 7002 mutant, missing genetic *A2687,* result of transformation with *A2687*::Ω	This study
Mut-A2697	Sp^r, *Synechococcus* 7002 mutant, missing genetic *A2697,* result of transformation with *A2697*::Ω	This study
Mut-A2833	Sp^r, *Synechococcus* 7002 mutant, missing genetic *A2833,* result of transformation with *A2833*::Ω	This study
Mut-F0050	Sp^r, *Synechococcus* 7002 mutant, missing genetic *F0050,* result of transformation with *F0050*::Ω	This study
Mut-F0106	Sp^r, *Synechococcus* 7002 mutant, missing genetic *F0106,* result of transformation with	This study

	F0106::Ω	
Mut-G0067-68	Sp^r, *Synechococcus* 7002 mutant, missing genetic *G0067* and *G0068,* result of transformation with *G0067-68*::Ω	This study
Mut-G0069-70	Sp^r, *Synechococcus* 7002 mutant, missing genetic *G0069* and *G0069,* result of transformation with *G0069-70*::Ω	This study

Table S4 Primers used in this study

Primier	Sequence (5'-3')
A0076-up-1	TCGACGCGTAACGCCACGGTTATGCCG
A0076-up-2	AGTGATATCCATAGGATTTACCTCTTG
A0076-dn-1	GGAAGATCTTAAATAAATTCACTGTTG
A0076-dn-2	CCGCTCGAGGCAAAACGAACTAAAGAA
A0079-up-1	CCGACGCGTTTCGCCTAGAAATCTCGA
A0079-up-2	AGTGATATCCATTCTGTTAGTTTAAAC
A0079-dn-1	AAACTGCAGTAGTGTGAAAGATAACAG
A0079-dn-2	CCGCTCGAGACTCGATGCCTAGTTTGG
A0148-50-up-1	CCGGAATTCAATTACTCTAGACATGGT
A0148-50-up-2	CGGTTTAAAGGGATTGTTGGCATACTT
A0148-50-dn-1	AAACTGCAGCCACCACCTCGTCAAGCC
A0148-50-dn-2	CCGCTCGAGATCGGGGACTGTTCCTGT
A0250-up-1	CCGGAATTCGGCAATGCAAAAACTATC
A0250-up-2	AGTGATATCCATAACGATAAAAACTCC
A0250-dn-1	AAACTGCAGTAGAGTTACAGGTTGAAT
A0250-dn-2	CCGCTCGAGATCAAAAAGTTTCAAGCA
A0340-up-1	CCGGAATTCTGGTACGTGGCCCTGGCG
A0340-up-2	AGTGATATCCATGGTGTCGCTCCTTTG
A0340-dn-1	AAACTGCAGTAAGCAGCGATACTGACA
A0340-dn-2	CCGCTCGAGTTGAAATCGCGGCCAAGA
A0424-up-1	CCGGAATTCACCCAATGACGCGGAACT
A0424-up-2	AGTGATATCCATGGTGATCGTGACTGG
A0424-dn-1	AAACTGCAGTGATTTTGATTTTTGCGC
A0424-dn-2	CCGCTCGAGGACCCGTTTCGGTTCGAT
A0479-up-1	CCGGAATTCTTTGCCCTTAACCCTCAG
A0479-up-2	AGTGATATCCATGGGCCTTCTCCGTCA
A0479-dn-1	AAACTGCAGTAAGTTGCTTTAAATTGG
A0479-dn-2	CCGCTCGAGGTCAATGATTTTAGTGAA
A0539-up-1	CCGACGCGTCAGGGGAATGGAGTTGCG
A0539-up-2	AGTGATATCCACAATCAGTTTGAACTC
A0539-dn-1	AAACTGCAGTAAGGGAATATTTAAACG
A0539-dn-2	CCGCTCGAGGTTTTCTCGCTACAGTGT
A0549-50-up-1	CCGGAATTCCAAAGATCGCCTTCGCCA

A0549-50-up-2	AGTGATATCCGATAACGGGCGGGATGC
A0549-50-dn-1	AAACTGCAGACGAAACCCTCTTTAGTT
A0549-50-dn-2	CCGCTCGAGGCATGCCCTCTTTGCTGA
A0556-up-1	CCGACGCGTTCGAAGTTCAAGCAAGCT
A0556-up-2	GCCTTTAAACACAGTTAATGCTCCTTT
A0556-dn-1	AAACTGCAGTAAGCGGCCCCAGAAAGT
A0556-dn-2	CCGCTCGAGAACCAGAACATGATGCTG
A0645-up-1	CCGGAATTCGGTCAAATTGGTGAGGCG
A0645-up-2	AGTGATATCCATGATCTCAGGGGGGAAAGAG
A0645-dn-1	AAACTGCAGTAATGTCAACTTCGGAAA
A0645-dn-2	CCGCTCGAGAATGGTTACCAACAGGAG
A0667-up-1	CCGGAATTCATTTCAGTTTGAACTGGC
A0667-up-2	AGTGATATCCACGCCAAGTATTTCGAT
A0667-dn-1	AAACTGCAGTAAATTCTATGCCAGCCG
A0667-dn-2	CCGCTCGAGAACTAATTGATTAGTCTG
A0685-up-1	CCGGAATTCGCTGCTCAAGCCCATCAG
A0685-up-2	AGTGATATCCATACAACGGCAGTCTAG
A0685-dn-1	GGAAGATCTTGAAAATTGCTATTCTCT
A0685-dn-2	CCGCTCGAGCTTGCACCAAAATGTTGG
A0709-up-1	CCGGAATTCACCTCACCACCAAAGTCG
A0709-up-2	AGTGATATCCATAACCGTGACAAAAAGCT
A0709-dn-1	GGAAGATCTTAAATGCCGCCCACAAAAC
A0709-dn-2	CCGCTCGAGGACTATTACAACGGTTAC
A0779-up-1	CCGGAATTCGCAACGGCGCCCACCACC
A0779-up-2	AGTGATATCCATCACTTACTGCACAGG
A0779-dn-1	AAACTGCAGTAGGGCTGGCTCCCTGCT
A0779-dn-2	CCGCTCGAGCAACGGCAGTACCGTACC
A0873-up-1	CCGGAATTCTTGAATTTGAGCCTGCTC
A0873-up-2	AGTGATATCCAAAAAAATAGCACTAATTCC
A0873-dn-1	AAACTGCAGTAGTTGTCAGTCAGACCT
A0873-dn-2	CCGCTCGAGGTCGAGTTGGTCACGGAA
A0892-93-up-1	CCGGAATTCGCGCTGAGTCATGGTGAA
A0892-93-up-2	AGTGATATCCATGATTGTAGGGTGCTT
A0892-93-dn-1	AAACTGCAGTAGACCGCCGCGATCCAT
A0892-93-dn-2	CCGCTCGAGTATTGCCCGGCGATCGCCA
A0922-up-1	CCGGAATTCGGCAAGTCTTTACCGTCT
A0922-up-2	AGTGATATCGACTTGATCAAGATTGAG
A0922-dn-1	AAACTGCAGAGCGATCTTTGTTGTATT
A0922-dn-2	CCGCTCGAGATTGCCAGGTTATCGGCT
A0960-up-1	CCGGAATTCGGGCTGCTTCGCCATATT
A0960-up-2	AGTGATATCCATCTTTTTTTTTCCTATGACC
A0960-dn-1	AAACTGCAGTGAGCGACCATCACATCA
A0960-dn-2	CCGCTCGAGTTTGAACACCAATTTCTG
A1077-up-1	CCGACGCGTAATGGCCAGAACCACCGC

A1077-up-2	AGTGATATCCATGATTTAGTTCCTTCT
A1077-dn-1	AAACTGCAGTAGGGACAGTCGTTAACA
A1077-dn-2	CCCAAGCTTTTTATGGTGTTGATTAGT
A1094-up-1	CCGACGCGTTTGGCCATTTTTGTGAAC
A1094-up-2	AGTGATATCCATGGCCAACCTCTTTTTGC
A1094-dn-1	AAACTGCAGTGAGGGGGCTTAAATTAA
A1094-dn-2	GGAAGATCTCCGCAAGGGGAATCGGCT
A1204-up-1	CCGACGCGTAGTGCTATGACTGAGGCA
A1204-up-2	GCGTTTAAACATTGATTACACTGCACA
A1204-dn-1	AAACTGCAGTAGTAAAGGTCATAAAAAAAGACG
A1204-dn-2	CCCAAGCTTAGCAATGGCCAAGCCGTG
A1232-up-1	CCGACGCGTATTAAGATCAAGAGTCAG
A1232-up-2	TTCCAGCTGCATGGGCTTACAAAAGTC
A1232-dn-1	AAACTGCAGTAGCGACTTGCCCTAACC
A1232-dn-2	GGAAGATCTTGCCATTGGCGCCATTGG
A1249-up-1	CCGACGCGTGTAATTGGGTCGGCACTA
A1249-up-2	GCGCAGCTGCAAGGGGCGTTTGTCTCC
A1249-dn-1	AAACTGCAGTAAATTTAGATTTTAAAAAC
A1249-dn-2	CCCAAGCTTGTTCTGGTTGGATCGAGA
A1320-up-1	CCGACGCGTTCGATAAATTCAAAGGGA
A1320-up-2	GCGTTTAAACATGATGAATGTTATCGA
A1320-dn-1	AAACTGCAGTAGTGGGTTTGAATTTAG
A1320-dn-2	CCCAAGCTTAAGGGCCACATCGCTGGC
A1339-up-1	CCGACGCGTTTTGAAAAAGGCCACCAG
A1339-up-2	GCGCAGCTGCATCAAAAAATAATGAGGTT
A1339-dn-1	AAACTGCAGTAAAGGGGCGATCGCCCGGAA
A1339-dn-2	GGAAGATCTAATGATCAATACCAACGC
A1360-up-1	CCGACGCGTTTAATGAACGGGCCTGGA
A1360-up-2	GCGTTTAAACATTCGCTACAAATATTC
A1360-dn-1	AAACTGCAGTAACGGCACTAGGGCAAG
A1360-dn-2	CCCAAGCTTATCTTTGGCGGGGTGGCCTA
A1429-up-1	CCGACGCGTCGAATCCGTCAGGACGAC
A1429-up-2	GCGTTTAAACATGGGAGGCTGACTCCT
A1429-dn-1	AAACTGCAGTGACGTTAAAAACTGAGC
A1429-dn-2	CCCAAGCTTACTCCGAAATTGCGCCAT
A1623-up-1	CCGACGCGTACGAGTTCGTCGTAGACA
A1623-up-2	AGTGATATCAGCCCCCGGCGATCGCCG
A1623-dn-1	AAACTGCAGTGGGAGATTCTGTCCATC
A1623-dn-2	CCCAAGCTTGTAATGGTGTGGTTTATT
A1895-up-1	CCGGAATTCATTACCCCCAGACAAGCT
A1895-up-2	GCGCAGCTGCATTGGAAAAGCAGCTTA
A1895-dn-1	GGAAGATCTTAATTTGAGAGAATGACT
A1895-dn-2	CCGCTCGAGCGTCCCCATCAACAATGC
A2101-up-1	CCGGAATTCATCTTTATGCCACAGGTA

A2101-up-2	AGTGATATCCAAAATCTATTGATTTAC
A2101-dn-1	AAACTGCAGTAGGGCCAGACTGATCGG
A2101-dn-2	CCGCTCGAGTACCAAGCCTGATAGGTG
A2284-86-up-1	CCGGAATTCGAACTGGTTTTTCGCGATT
A2284-86-up-2	AGTGATATCCATAGTTTCCTCTATTGT
A2284-86-dn-1	AAACTGCAGTAAAGCTCTAAAACAAAATTG
A2284-86-dn-2	CCGCTCGAGCTTTGAAGGGGCAGTGAA
A2347-up-1	CCGGAATTCCTGCCCTATGAGGTGGTC
A2347-up-2	AGTGATATCCATGGGATTGCGTGCTCC
A2347-dn-1	AAACTGCAGTAGGCCAGAGGATGCTGG
A2347-dn-2	CCGCTCGAGTCGGTGGGTAAAACGGTT
A2352-up-1	CCGGAATTCGCAAAGGAACCGCGCCAG
A2352-up-2	AGTGATATCTTGGGGCGGAAAGATTAG
A2352-dn-1	AAACTGCAGGTTCGGGCCAATCAAGAG
A2352-dn-2	CCGCTCGAGCTGCGTTCTATTGTTCAA
A2472-73-up-1	CCGGAATTCCTATTGGGCGATTTATCG
A2472-73-up-2	AGTGATATCCATGGTCTAGGCTTGGGT
A2472-73-dn-1	AAACTGCAGTAATCTCCCCTTAAAACC
A2472-73-dn-2	CCGCTCGAGAGGTACAGAGGTTTAATTC
A2598-99-up-1	CCGGAATTCGATTTTGCCATGGTCGCT
A2598-99-up-2	AGTGATATCCAAAACTTACTGATTGAC
A2598-99-dn-1	AAACTGCAGTAACCGCAAACCAAGATT
A2598-99-dn-2	CCGCTCGAGTGAAACAGAAACGGCCTA
A2602-up-1	CGCGTCGACCATCCAAGAACGCCTCGA
A2602-up-2	AGTGATATCCATATGAAACACACTAGC
A2602-dn-1	GGAAGATCTTAGAGGTTGTATTTCATA
A2602-dn-2	CCGCTCGAGACAGTTGAACCCGGAAGA
A2687-up-1	CGCGTCGACGGCCGTTGAAACGCTCCA
A2687-up-2	TAACAGCTGCATGGCTTAGGGCCGCCT
A2687-dn-1	AATGGGCCCTAACTCTGGTATCACTTC
A2687-dn-2	AACGAGCTCTTTGGCGGATCTGGCTGC
A2697-up-1	CGCGTCGACTTAGCGTCGGGGGAGACA
A2697-up-2	TAACAGCTGCATATATTTGTGCGTCAG
A2697-dn-1	AATGGGCCCTAAGGGAACCTCGCTTAA
A2697-dn-2	AACGAGCTCCAGGGTCGTGCTGAGTCC
A2833-up-1	CGCGTCGACGTAATGATGGCGATCGCC
A2833-up-2	TAACAGCTGCAAAATGCTTTCCTAACG
A2833-dn-1	AATGGGCCCTAGATTGTTGTCCCCAAA
A2833-dn-2	AACGAGCTCGAAGCCTACCGCCTGCAA
F0050-up-1	CGCGTCGACGGTGCCCTTGTGGGACTC
F0050-up-2	CGGTTTAAACATTTTTAGTACAAACAA
F0050-dn-1	GGAAGATCTTGACCTGGCACTATGATG
F0050-dn-2	CCGCTCGAGAAGTCATTTATTCATTAT
F0106-up-1	CGCGTCGACCTGAAGGAGCTGGTCTAC

F0106-up-2	TAACAGCTGCATAAAGAAGACCTCTAT
F0106-dn-1	GGAAGATCTTAGGACTTTTTATCATGAAC
F0106-dn-2	CCGCTCGAGGAGGAACTCTCTGGCAGC
G0067-68-up-1	CGCGTCGACTGAAGTCCTTGTATTGTG
G0067-68-up-2	TAACAGCTGTAGAGCAGACGTATTGGA
G0067-68-dn-1	GGAAGATCTTAGCAATACATTAATTTA
G0067-68-dn-2	CCGCTCGAGGCGCATAATAATCACTGA
G0069-70-up-1	CGCGTCGACTTAAACAAGTTAGGTGGG
G0069-70-up-2	TAACAGCTGCAAAATAGGGAAAAATAATTAAG
G0069-70-dn-1	AATGGGCCCTAACTGAGCTAAAAAAAAACTTTAG
G0069-70-dn-2	TTCGAGCTCATGAAGGAGGGTTTATGG

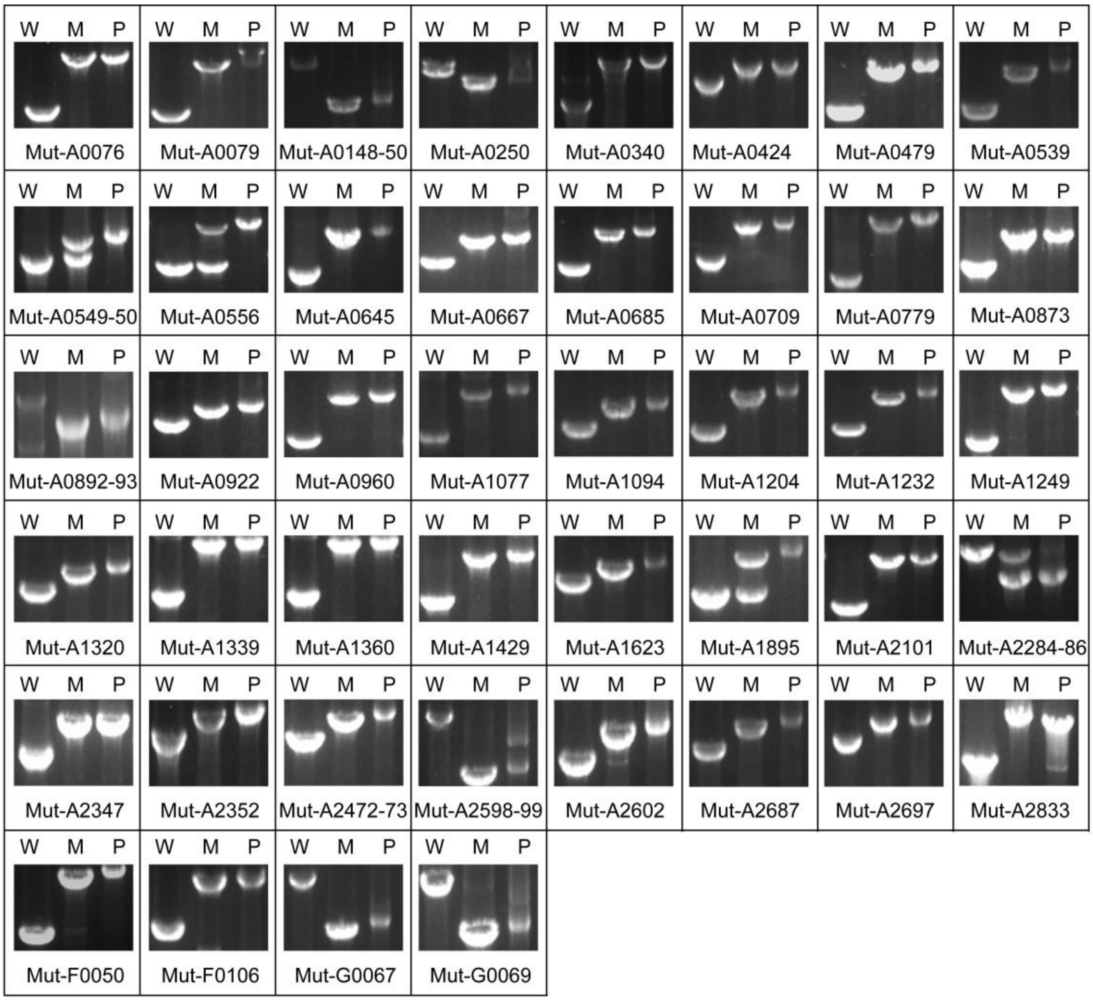

Fig. S1. PCR detection of 44 mutant strains. W is the genome of the wild-type algal strain, M is the genome of the mutant, and P represents the use of the recombinant plasmid

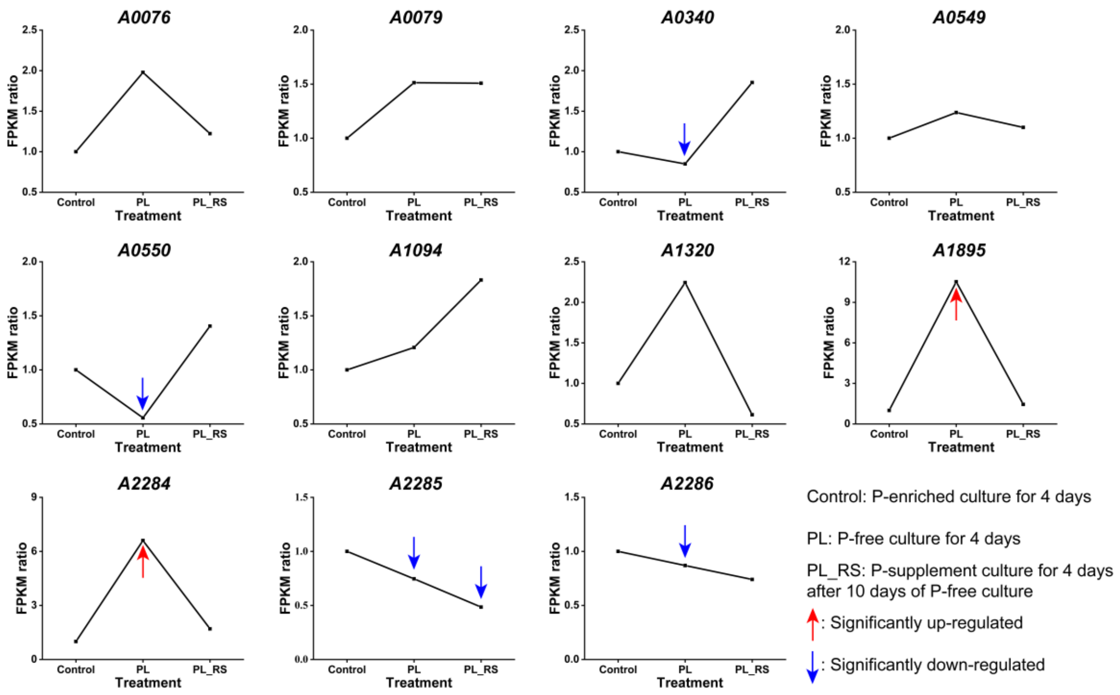

Fig. S2. Map of changes in FPKM ratio of genes. Ordinate values are the FPKM ratio of the gene under PL or PL_RS treatment to the FPKM of the Control. Arrows represent significant changes in transcript levels of this gene under this treatment. Red arrows represent significant up-regulation and blue arrows represent significant down-regulation.

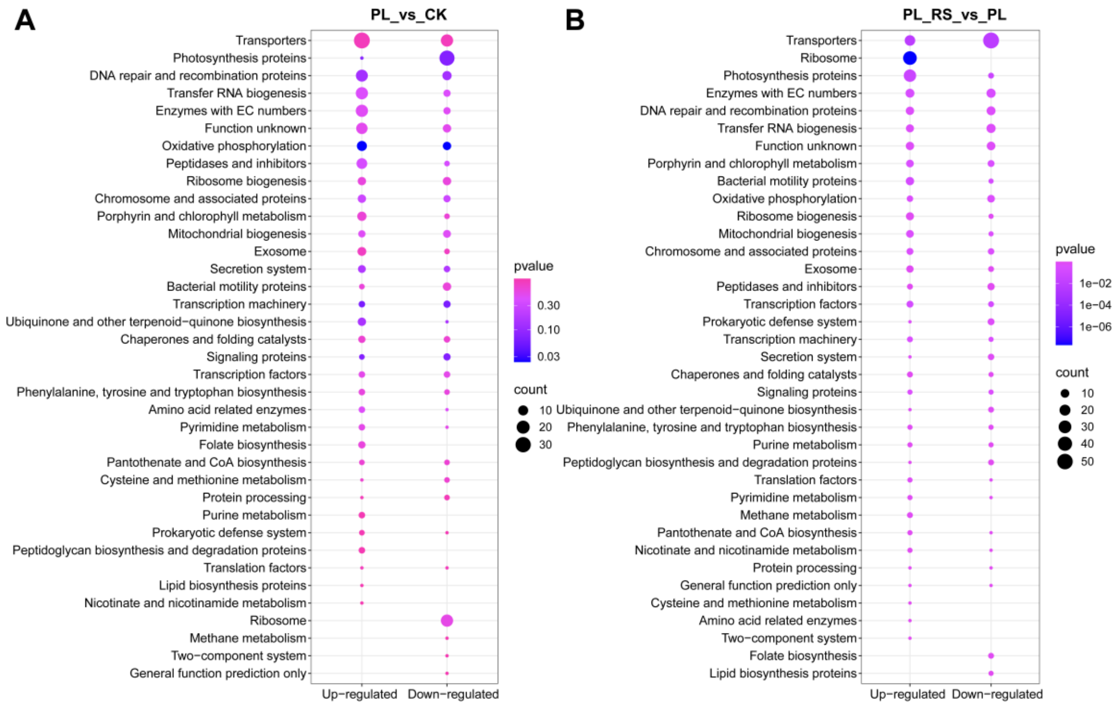

Fig. S3. Analysis of up-regulated and down-regulated DEGs among different treatment groups. A and B KEGG enrichment analysis during P deficiency and P recovery. The size of the dot depends on the number of genes enriched in the pathway, and the color of the dot indicates the significance of pathway enrichment.

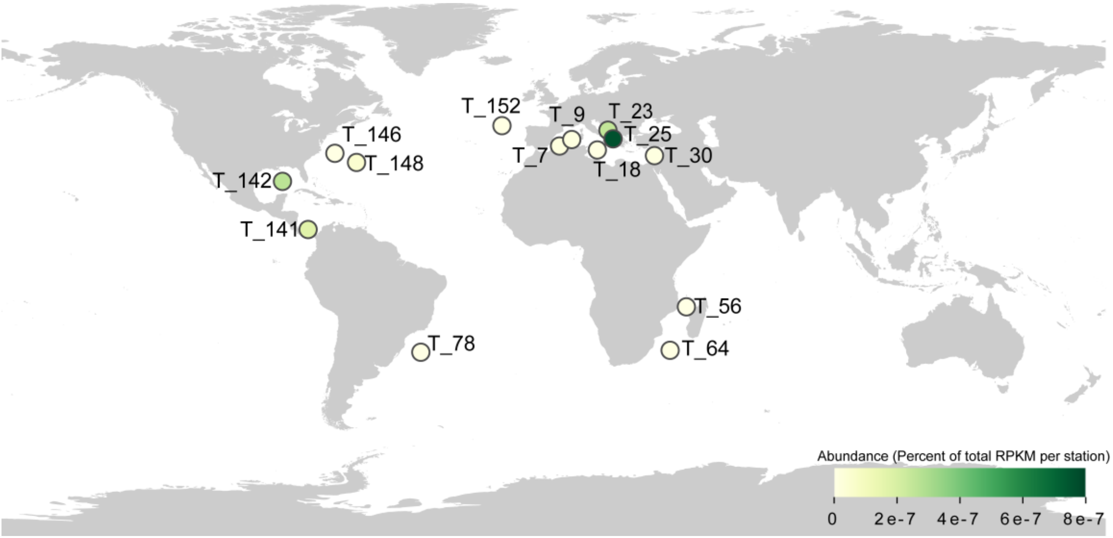

Fig. S4. Distribution of *Synechococcus* sp. PCC 7002 in the ocean around the world. The dot in the figure represents the *phoU* of 7002 detected at this site. The distribution of *phoU* in *Synechococcus* sp. PCC 7002 was explored using the Tara Oceans Microbiome Reference Gene Catalog ((http://tara-oceans.mio.osupytheas.fr/Ocean-gene-atlas/)) database. The expectation threshold was set to 1E-8.
